# It is not all about the alpha: elevated expression of p53β variants is associated with lower probability of survival in a retrospective melanoma cohort

**DOI:** 10.1186/s12935-023-03083-6

**Published:** 2023-10-04

**Authors:** Kira Groen, Luiza Steffens Reinhardt, Jean-Christophe Bourdon, Kelly A. Avery-Kiejda

**Affiliations:** 1 School of Biomedical Sciences and Pharmacy, College of Health, Medicine and Wellbeing, The University of Newcastle, Callaghan, NSW Australia; 2 Hunter Medical Research Institute, Level 3 West, Lot 1 Kookaburra Circuit, New Lambton Heights, NSW Australia; 3 Cancer Detection & Therapy Research Program, Hunter Medical Research Institute, New Lambton Heights, NSW Australia; 4 School of Medicine, Ninewells Hospital and Medical School, The University of Dundee, Dundee, UK

**Keywords:** p53 isoforms, Melanoma, Immunohistochemistry, Biomarker

## Abstract

**Background:**

Melanoma is the deadliest type of skin cancer and despite improvements in treatment outcomes, melanoma claimed 57,043 lives in 2020. In most malignancies, p53 mutation rates are above 50% and provide prognostic indications. However, in melanoma where less than a quarter of cases harbour a p53 mutation, the significance of the tumour suppressor may be questioned. Instead, p53 isoforms, which modulate p53’s canonical function, may be of greater clinical importance.

**Methods:**

The expression of p53 isoforms was evaluated in 123 melanoma specimens by immunohistochemistry using p53 isoform-specific antibodies (DO-1, KJC8, KJC40, and KJC133). To determine whether *TP53* mutations may be driving p53 isoform expression, *TP53* was sequenced in 30 FFPE melanoma samples.

**Results:**

The C-terminally truncated p53β isoforms (KJC8) were found to be the most highly expressed p53 isoforms compared to all other isoforms. Further, elevated KJC8 staining was found to correlate with reduced probability of melanoma-specific survival, while KJC40 staining (Δ40p53) positively correlated with reduced melanoma thickness. TAp53 isoforms (p53 retaining both transactivation domains, DO-1), were the second highest p53 isoforms expressed across all samples. Elevated DO-1 staining was also associated with worse survival outcomes and more advanced stages of cancer. Given that the isoforms are likely to work in concert, composite isoform profiles were generated. Composite biomarker profiles revealed that elevated TAp53 (DO-1) and p53β (KJC8) expression, accompanied by low Δ40p53 (KJC40) and Δ133p53 (KJC133) expression was associated with the worst survival outcomes. Supporting the lack of predictive biomarker potential of *TP53* in melanoma, no clinicopathological or p53 isoform expression associations could be linked to *TP53* status.

**Conclusions:**

Given the lack of prognostic biomarker potential derived from *TP53* status, this study highlights how p53 isoform expression might progress this field and, pending further validation, may provide additional information to treating oncologists that might be factored into treatment decisions.

**Supplementary Information:**

The online version contains supplementary material available at 10.1186/s12935-023-03083-6.

## Background

Melanoma is the most aggressive type of skin cancer. While the advent of targeted therapies (i.e., *BRAF* and *MEK* inhibitors) and immune checkpoint inhibitors (i.e., ipilimumab, pembrolizumab, nivolumab) significantly improved recurrence and survival outcomes for melanoma patients, metastatic melanoma and therapy resistance still represent major challenges for clinicians [1]. Currently, there is no reliable prognostic marker for melanoma.

p53, also known as the “guardian of the genome”, is a critical tumour suppressor that plays a role in cell cycle regulation, DNA repair, senescence, and apoptosis, through transactivation of target genes and protein interactions with key components of cellular pathways [2]. As such, *TP53* has been found to be mutated in around half of all human cancers. However, its mutation rate in melanoma is much lower than the average, sitting below 20% (cBioPortal database for cancer genomics [3]). With the importance p53 plays in cancer suppression and maintenance of genome integrity [2], it is likely that its pathway activity is deregulated even in the absence of mutations. In fact, previous research from our laboratory has shown that p53’s transcriptional activity is deregulated in metastatic melanoma samples and cell lines regardless of p53 mutation status and that p53 may in fact contribute to proliferation of tumour cells, rather than contributing to their senescence and apoptosis [4]. Other groups have also confirmed that wild-type p53 fails to act as a tumour suppressor in melanoma [5, 6]. Several hypotheses that may explain impaired p53 pathway activity in p53 wild-type melanoma have been proposed, including mutations of *CDKN2A* (encoding p14^ARF^, which inhibits p53 degradation by HDM2) and overexpression of HDM2 (human double minute 2) or anti-apoptotic proteins (iASPP and BCL-2) [7]. Yet, the induction of wild-type p53 in melanoma in response to genotoxic stress suggests regulatory mechanisms upstream of p53 remain intact at least in some cases [5, 8]. Further, intrinsic apoptotic mechanisms have been found to be operational in melanoma [9], negating the hypothesis for overexpression of anti-apoptotic proteins as the driving mechanism for p53’s inability to function as a tumour suppressor in melanoma. An alternative hypothesis is that the aberrant regulation of p53 pathway activity in melanoma may be driven by the differential expression of p53 isoforms. However, this has not been extensively studied.

p53 may be expressed as full-length p53 (p53α from herein) or as 12 shorter isoforms bearing a combination of N- (Δ40p53, Δ133p53, Δ160p53) and C-terminal (β, γ, Ψ) truncations. These isoforms may be generated through alternative splicing (Δ40p53, p53β, p53γ), alternative promoter usage (Δ133p53, Δ160p53), alternative initiation of translation (Δ40p53, Δ160p53), or post-translational degradation of p53 via the 20S proteasome (Δ40p53) [10]. The isoforms have been found to be aberrantly expressed in various cancers, including breast cancer [11, 12], squamous cell carcinoma of the head and neck [13], and neuroblastoma [14] among others. p53 isoforms are known to both enhance and inhibit p53’s canonical function in a cell- and context-specific manner [14–17], highlighting their potential to contribute to dysregulated p53 pathway activity in melanoma.

Previous data from our laboratory highlighted that p53β and Δ40p53 were expressed at both the mRNA and protein level in melanoma cell lines and primary melanoma cultures, while undetectable or expressed at low levels in normal melanocytes and fibroblasts [17]. Contrastingly, in metastatic melanoma Δ40p53β mRNA expression was found to be lower in tumour compared to normal adjacent tissue, while Δ133p53α and Δ160p53α protein expression was greater compared to normal adjacent tissue. However, gene expression findings did not always correspond with protein findings and vice versa [18, 19]. In melanoma cell lines, exogenous p53β was found to enhance p53 target gene expression in response to chemotherapeutic treatment with cisplatin, while Δ40p53 was found to impair the upregulation of the same p53 target genes following cisplatin treatment [17]. However, Takahashi and colleagues reported that exogenous Δ40p53 enhanced apoptosis in cancerous and noncancerous cells, though this was in the absence of chemotherapeutic agents [20] and supports previous data from our laboratory, showing that at the basal level Δ40p53 acts similarly to p53α and functions as a tumour suppressor [21]. Δ160p53 isoforms have also been found to promote proliferation and possibly migration when transfected into melanoma cell lines [19]. Further, endogenous p53 isoforms have been linked to treatment resistance in melanoma cell lines, with BRAF-inhibitor resistant cell lines harbouring increased expression of Δ40p53β and decreased expression of TAp53β [19].

Thus, there is evidence of p53 isoform deregulation in melanoma cell lines and metastatic melanoma [17, 18], and in vitro studies suggest that p53 isoforms may affect melanoma aggressiveness and treatment response [17, 20]. Altogether this indicates that p53 isoforms may harbour biomarker potential in melanoma. However, studies of p53 isoforms in primary melanoma specimens are still lacking and this represents a crucial next step to using p53 isoforms expression in a clinical setting. Melanomas are frequently preserved through formalin fixation and paraffin embedding, which impedes the assessment of p53 isoforms at the mRNA level due to nucleic acid degradation [22]. In the present study we investigated the expression of p53 isoforms by immunohistochemistry (IHC), using a suite of C- and N-terminal p53 isoform-specific antibodies in a retrospective melanoma cohort and evaluated their prognostic biomarker potential.

## Methods

### Study cohort

The study cohort comprised formalin-fixed, paraffin-embedded (FFPE) sections from 71 primary melanoma cases and 52 metastatic melanoma cases, including 20 matched primary and metastatic samples and five pairs of metastatic melanomas obtained from the same individual. Cohort characteristics are summarised in Additional file 1: Table S1. FFPE melanoma blocks were cut into 4 μm sections and mounted onto coated slides. Slides were obtained from the NSW Regional Biospecimen and Research Service (Newcastle, NSW, Australia), who obtained the tissue samples in accordance with the Declaration of Helsinki and whose operations have been approved by the Hunter New England Human Research Ethics Committee (HNEHREC) (Reference Number: 12/06/20/5.03). Ethics approval for this project was also obtained from the HNEHREC (Approval Number: 2020/ETH00251).

### Antibodies

**Fig. 1:**
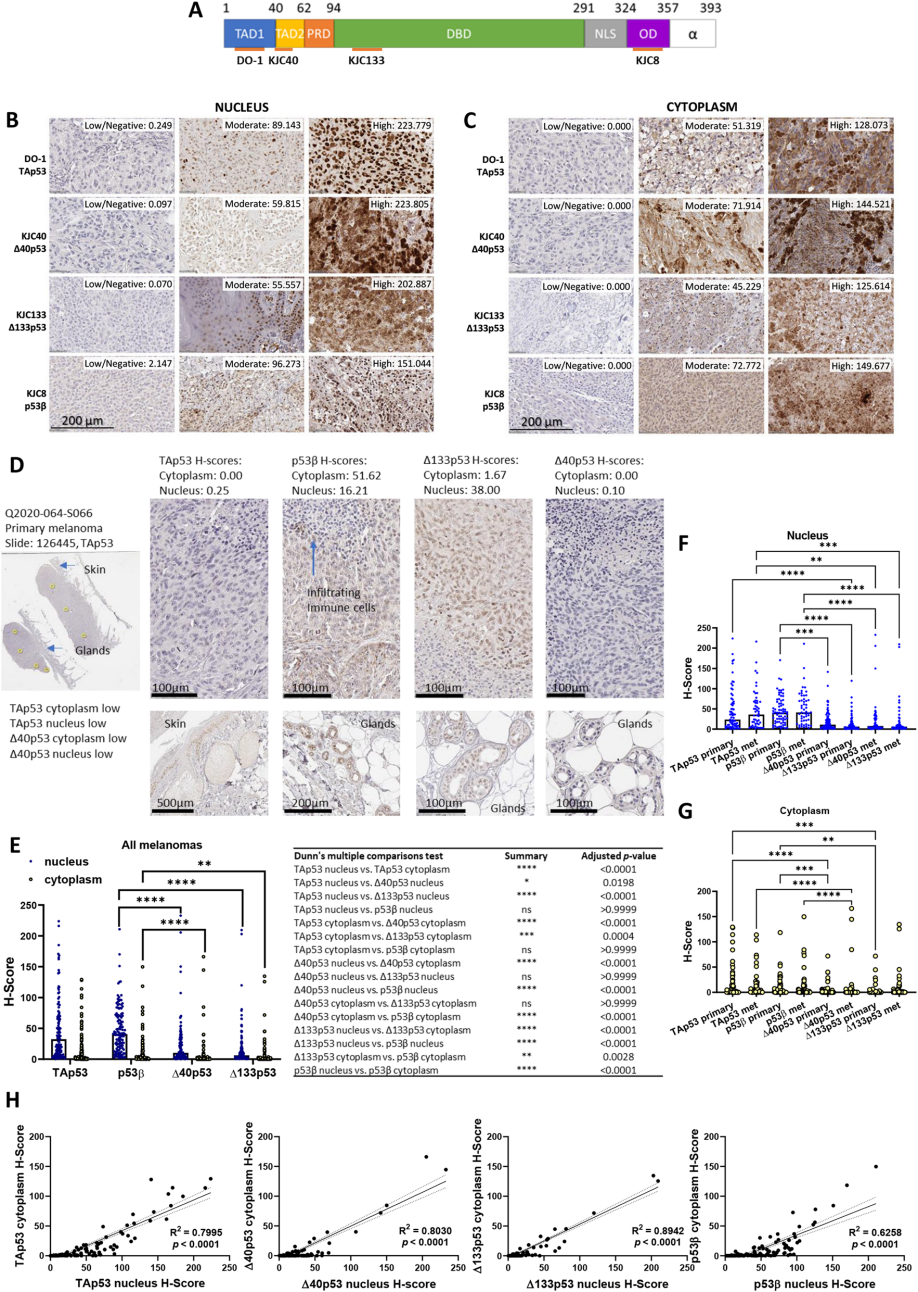
p53β is the most highly expressed p53 isoform in melanoma. **A**. p53 functional domains, C- and N-terminal isoforms assessed by antibodies in orange. Numbers indicate amino acid position from the start codon. TAD– transactivation domain; PRD– proline-rich domain; DBD– DNA binding domain; NLS– nuclear localisation signal; OD– oligomerisation domain. **B**. Nuclear and **C**. cytoplasmic expression of p53 and its isoforms in melanomas stained (brown) with DO-1 (TAp53), KJC40 (Δ40p53), KJC133 (Δ133p53), and KJC8 (p53β). Representative images of low, median, and high expression in 123 melanomas. Associated H-scores displayed next to images. **D**. Staining of a primary melanoma with DO-1 (TAp53), KJC8 (p53β), KJC40 (Δ40p53), and KJC133 (Δ133p53). The same sample was used in Fig. 1A and B to illustrate low TAp53 and low Δ40p53 staining in the nucleus and cytoplasm. H-scores for each antibody are shown above images. Yellow circles (n = 7) indicate areas used to quantify expression and generate H-scores. Other areas of interest are indicated by blue arrows (normal skin, glands, and infiltrating immune cells). Zoomed in pictures of normal skin, and glands are shown. Scale bars as indicated. Additional samples showing area selection, staining of all antibodies in the same sample, and other structures of interest can be found in Additional file 5. **E**–**G**. TAp53 (DO-1), p53β (KJC8), Δ40p53 (KJC40), and Δ133p53 (KJC133) H-Scores measured in nucleus and cytoplasm of **E**. all melanomas (primary and metastasis combined, n = 123) and **F**, **G** primary (n = 71) and metastatic (n = 52) melanomas separately. **H**. Correlations between nuclear and cytoplasmic p53 isoforms in melanoma with R^2^ values and *p*-values of the linear regression displayed. Significance determined through Friedman test (matched **E**) or Kruskal–Wallis test (unmatched **F** & **G**), corrected for multiple comparisons with Dunn’s multiple comparisons test. For **E** only some *p*-values are displayed, with all *p*-values shown in the adjacent table. *p < 0.05, **p < 0.01; ***p < 0.001; ****p < 0.0001

The following monoclonal antibodies were used for IHC: mouse monoclonal DO-1 (epitope within transactivation domain of p53, TAp53, detects p53α, p53β, and p53γ), and rabbit polyclonal antibodies KJC8 (detects the p53β isoforms) [15, 23], KJC40 (detects the Δ40p53 isoforms, mainly Δ40p53α) [21], KJC133 (detects the Δ133p53 isoforms) [12] (Fig. 1A). DO-1, KJC8, KJC40, and KJC133 antibodies were provided from the University of Dundee (antibodies were developed by Dr. Jean-Christophe Bourdon, Dundee, Scotland, United Kingdom).

### Immunohistochemistry

IHC was performed by the NSW Regional Biospecimen & Research Service (Newcastle, NSW, Australia) using the Ventana Discovery Automated Immunostainer (Roche Medical Systems, Tucson, AZ, USA) as previously described [12]. After antigen retrieval [12], sections were pre-treated overnight at room temperature with 10% H_2_O_2_ phosphate buffered saline (pH 7.4) to bleach the samples. Following bleaching, slides were incubated for 12 min with peroxidase inhibitor (Roche Medical Systems). Next, tissue sections were incubated with primary antibodies at a dilution of 1:160 (DO-1), 1:40 (KJC8 and KJC40), or 1:100 (KJC133) for 32 min at 37 °C. Slides were then incubated with secondary and tertiary antibodies as described [12] and the immunolocalised isoforms were visualised using a DAB chromogen detection kit (Roche Medical Systems). All slides were counterstained, rinsed and dehydrated as described [12]. Slides were then sealed with a glass coverslip and allowed to dry. Slides were scanned at 40 × magnification using an Aperio AT2 scanner (Leica, Wetzlar, Germany). Immunostained slides were histologically evaluated by an expert pathologist and analysed with HALO Software (Halo imaging analysis software, Indica Labs, Corrales, NM, USA) using the CytoNuclear v2.0.8 analysis mode, which automatically scores the staining intensity from weak to strong. Annotations were manually selected to represent the tumour tissue and to exclude tissue artefacts. H-scores for each p53 isoform were quantified for each tumour in both the nucleus and cytoplasm.

### *TP53* sequencing

#### DNA extraction

Thirty primary melanoma samples were selected for *TP53* sequencing. Samples were chosen to represent low TAp53 expression (n = 10), moderate TAp53 expression (n = 10), and high TAp53 expression (n = 10) by ranking all primary melanoma samples according to TAp53 expression and selecting the top, middle, and bottom 10 samples. DNA was extracted from three 10 μm tissue scrolls with the Zymo Research Quick DNA FFPE Mini kit (Integrated Sciences, Chatswood NSW, Australia). DNA was quantified with the Qubit fluorometer using dsDNA Broad Range Assay kit (ThermoFisher Scientific, Socresby VIC, Australia) as per the manufacturer’s protocol. Extracted DNA was stored at -80 °C until library preparation. One sample was excluded prior to library preparation as insufficient DNA was extracted (< 50 ng).

#### Library preparation

Next-generation sequencing libraries were prepared from 112.5 ng genomic DNA with an AmpliSeq PLUS kit (Illumina, Singapore, Singapore), using an AmpliSeq Custom DNA Panel (Illumina, San Diego, CA, USA) for *TP53* (Additional file 2, 3: Table S2-S3; exons only, 98.88% coverage, amplicons < 140 bp due to potential fragmentation from formalin-fixing, paraffin embedding process) as per the manufacturer’s protocol. Target amplification, using Illumina provided primers, was performed on a Veriti Thermal Cycler (Applied Biosystems, ThermoFisher Scientific, Socresby VIC, Australia). Amplicons were partially digested with the provided FuPa reagent (Illumina) before the index ligation reaction was carried out on a Veriti Thermal Cycler (Applied Biosystems). Libraries were cleaned up using AMPure XP Beads (Beckman Coulter, IN, USA), amplified on a Veriti Thermal Cycler and cleaned up again using AMPure XP Beads (Beckman Coulter) as per Illumina standard protocol. Libraries were checked for size on an Agilent Tapesation with Agilent D1000 Screen Tape and Reagents (Integrated Sciences, Chatswood NSW, Australia) and concentration was determined with the Qubit fluorometer using dsDNA High Sensitivity Assay kit (ThermoFisher Scientific, Socresby VIC, Australia) as per the manufacturer’s protocol. Two samples were excluded due to low library yields (< 2 nM).

#### Sequencing

Twenty-seven libraries were pooled and sequenced on an Illumina MiSeq with a MiSeq Reagent Micro Kit v.2 (300 cycles, paired end).

Bioinformatic Analysis: Quality control was performed using MultiQC [24]. All 27 samples passed QC and were subjected to Illumina DNA Amplicon App v. 2.1.1 (Illumina, San Diego, CA, USA) analysis using the *Homo sapiens* UCSC hg19 as the reference genome, Ensembl as the annotation source, and setting the depth threshold to 10.

### Statistical analysis

G*Power 3.1 [25] was used to perform calculations on sample size, effect size, and statistical power. The minimal significance (α) and statistical power (1-β) were set at 0.05 and 0.80, respectively. The minimum sample size to achieve significance was calculated to be 42 samples per group, given the use of retrospective cohort, all available samples were included (n = 123). Parametric distribution was assessed through Shapiro–Wilk Tests. All IHC and continuous cohort data was found to follow a non-parametric distribution that could not be corrected with log-transformation and is thus represented as median and interquartile range (IQR). Differences in cohort characteristics between primary and metastatic cases were assessed by Whitney U Test (age) or Fisher’s exact test (Sex, *BRAF* and *TP53* mutation status). Differences between multiple groups of matched data (comparisons between IHC scores of matched primary and metastatic samples) were evaluated through Friedman test, while differences between multiple groups of unmatched data (comparisons between IHC scores of unmatched primary and metastatic samples) were assessed through Kruskal–Wallis test, both were corrected for multiple comparisons using the Dunn’s multiple comparisons test. Correlations between nuclear and cytoplasmic isoform expression and between expression of different isoforms within the same melanoma were evaluated using simple linear regression. Correlation between isoform expression and clinical parameters were determined through Spearman’s rho correlation analysis. Survival analysis for melanoma-specific survival and development of metastasis was evaluated using Kaplan–Meier analysis and differences in survival curves were determined by Log-rank (Mantel-Cox test). The hazard ratio (HR) and the 95% of confidence interval (CI) were also determined by Log-rank. All statistical analysis was carried out in GraphPad Prism (Version 9) (GraphPad Software, La Jolla, CA). An adjusted *p*-value of < 0.05 was deemed statistically significant. Cut-off values for survival analysis were determined using Cutoff-finder [26] and cut-off values for combined isoform profiles were created using median expression as a cut-off. For correlations with clinical analysis only one of the matched samples (primary melanoma for matched primary and metastatic cases, and first listed metastatic melanoma for matched metastatic cases) was used. All *p*-values were adjusted for multiple comparisons using the Benjamini–Hochberg procedure using a 10% false discovery rate. Given the exploratory nature of the study, all findings were reported regardless of whether they passed multiple correction testing, however this was noted in the results and figure legends.

## Results

The expression of p53 isoforms was investigated by IHC using a suite of p53 isoform-specific antibodies (Fig. 1A) in a retrospective melanoma cohort of 71 (57.72%) primary melanoma samples and 52 (42.28%) metastatic melanoma samples. The median age at diagnosis was 71 years old (range 21–98 years) and 34.15% of melanoma samples were obtained from female patients. There were no significant differences in clinical or demographic characteristics between the primary and metastatic melanoma cases. The clinical characteristics of the melanoma specimens are summarised in Additional file 1: Table S1.

By segregating the specimens based on the H-scores, it was possible to observe that the p53 isoform expression varied considerably among samples, ranging from low or negative to strong staining (Fig. 1B, C and E). Moreover, the subcellular localisation of the isoforms varied, with some specimens showing predominantly nuclear staining for all p53 isoforms (Fig. 1B), and others showing primarily cytoplasmic staining (Fig. 1C). In some instances, normal appearing skin on the margins of the melanoma stained positive for TAp53 isoforms as detected by DO-1 and Δ40p53 isoforms as detected by KJC40, but not p53β isoforms detected with KJC8 or Δ133p53 isoforms detected with KJC133. Contrastingly, glands in the subcutaneous fat layer stained positive for KJC8 (p53β). Infiltrating lymphocytes did not stain positive for any of the assessed p53 isoforms. Generally staining was stronger in the tumour tissue compared to normal appearing adjacent tissue (Fig. 1D and Additional file 5). These results indicate that p53 isoforms are potentially expressed in a highly context- and cell-specific manner [27] and may thus harbour biomarker potential. Staining in normal surrounding tissue may indicate upregulation of tumour suppressor pathways in these tissues [12], while elevated p53β and Δ133p53 in melanoma may be linked to cancer biology. In fact, Δ133p53β has been associated with cell invasiveness and cancer recurrence in luminal A breast cancer [28]. However, these findings would need to be confirmed in melanoma and the hypotheses validated.

### C-terminally truncated p53β isoforms are the most highly expressed p53 isoforms in melanoma

In all melanomas, the C-terminally truncated p53β isoforms were the most highly detected p53 isoforms in the nucleus (median H-score: 40.81, IQR: 50.63), followed by TAp53 isoforms (median H-score: 32.50, IQR: 59.63, not significant), and the N-terminally truncated Δ40p53 isoforms (median H-score: 10.34, IQR: 25.68, *p* < 0.0001) and Δ133p53 isoforms (median H-score: 6.28, IQR: 13.15, *p* < 0.0001). This challenges previous assumption that p53α is the most abundant isoform encoded by *TP53* [15, 29, 30]. In the cytoplasm, the expression of all p53 isoforms was significantly lower than in the nucleus (*p* < 0.0001) and the most highly detected isoforms were TAp53 (median H-score: 1.30, IQR 13.29), followed closely by p53β isoforms (median H-score: 1.01, IQR: 9.41, ns), Δ133p53 isoforms (median H-score: 0.15, IQR 1.18, *p* < 0.001), and Δ40p53 isoforms (median H-score: 0.03, IQR: 0.70, *p* < 0.0001) (Fig. 1E). Looking at primary and metastatic melanomas separately, the trend of expression remains the same with p53β isoforms being the most highly detected nuclear isoforms and TAp53 being the most highly detected cytoplasmic isoforms. There were no significant differences in expression of the same isoforms between primary and metastatic samples (Fig. 1F and G).

For all p53 isoforms nuclear expression levels were correlated with cytoplasmic expression levels, indicating that elevated nuclear expression of the p53 isoforms was typically accompanied by elevated cytoplasmic expression of the same isoform. The strongest correlation was observed between nuclear and cytoplasmic Δ133p53 H-scores (R^2^ = 0.89, *p* < 0.0001), while nuclear and cytoplasmic p53β H-scores were the least correlated (R^2^ = 0.63, *p* < 0.0001) (Fig. 1H).

In metastatic samples, p53 isoform expression generally did not vary between metastatic sites (Additional file 1: Fig. S1A and B) apart from cytoplasmic Δ40p53 isoforms which were found to be lowly detected (*p* < 0.05) in lymph node metastases (median H-score: 3.44, IQR: 7.01) but almost exclusively not detected in skin metastases (median H-score: 0, IQR: 0.034) (Additional file 1: Fig. S1B).

Correlation between primary and metastatic staining patterns supports the use of staining of primary tumours as biomarkers for metastases that might be harder to access, such as brain metastasis, while different subcellular localisation may indicate different functions of the p53 isoforms [12, 15, 16, 31]. While in the nucleus p53 isoforms are known to either enhance or inhibit canonical p53 transcriptional activity, their function in the cytoplasm remains poorly understood and warrants further investigation [15].

### p53β isoforms correlate with detection of N-terminally truncated isoforms in the cytoplasm

As C- and N-terminal isoforms can coexist, we examined the relationship between the expression of different isoforms to evaluate potential co-expression patterns. Nuclear p53β detected by KJC8 did not correlate significantly (R^2^ < 0.3) with any of the assessed N-terminal isoforms (TAp53, Δ40p53, or Δ133p53). However, in the cytoplasm p53β H-scores were found to correlate with both shorter N-terminal isoforms (Δ40p53 R^2^ = 0.343, *p* < 0.0001; Δ133p53 R^2^ = 0.383, *p* < 0.0001), but not TAp53 (R^2^ < 0.3) (Additional file 1: Fig. S2A). Δ40p53 detection was found to correlate with Δ133p53 (R^2^ = 0.524, *p* < 0.0001) in the nucleus and with the detection of both Δ133p53 (R^2^ = 0.698, *p* < 0.0001) and TAp53 (R^2^ = 0.397, *p* < 0.0001) in the cytoplasm (Additional file 1: Fig. S2B). Δ133p53 detection did not correlate with TAp53 detection in the nucleus nor the cytoplasm (Additional file 1: Fig. S2C), supporting previous observations that Δ133p53 may have p53-independent activities and regulation (reviewed in [29, 32]). While different N-terminal truncations cannot be found on the same protein, these relationships provide clues to potential co-expression, possibly through shared regulatory mechanisms. Additionally, some p53 isoforms may affect the subcellular localisation of others, for instance Δ40p53 is known to be associated with full-length p53 monoubiquitination and subsequent nuclear export [16], which may underpin the correlation between cytoplasmic TAp53 and Δ40p53. This has functional implications and may be a mechanism by which Δ40p53 impairs p53’s transcriptional activity [16, 31, 33] rendering the tumour suppressor’s transactivational capacity inactive in the absence of mutations. Further research is needed to identify the potential reasons underpinning other p53 isoform correlations.

Our previous research indicates that it is not solely the expression of p53 isoforms but their expression relative to canonical full-length p53 that is of clinical importance [11]. As no monoclonal antibodies can target both C- and N-terminal regions of p53 simultaneously (Fig. 1A), it was not possible to assess the expression of p53 isoforms relative to the canonical full-length p53α protein. This has thus far only been achievable through Western blotting, where both antibody staining and protein size can be used to identify isoforms and the full-length protein. Here we used TAp53 detection by DO-1 as a surrogate for canonical full-length p53, to compare with the expression of the p53 isoforms. The nuclear p53β:TAp53 ratio (KJC8/DO-1, median: 1.26, IQR: 2.70) was found to be greater than the nuclear Δ40p53:TAp53 ratio (KJC40/DO-1, median: 0.51, IQR 1.12, *p* < 0.0001) and the nuclear Δ133p53:TAp53 ratio (KJC133/DO-1, median: 0.27, IQR: 0.86, *p* < 0.0001) (Additional file 1: Fig. S3A). Similar results were observed when looking at cytoplasmic expression ratios of the shorter p53 isoforms (Additional file 1: Fig. S3A) and expression of primary and metastatic melanomas independently (Additional file 1: Fig. S3B & C). The cytoplasmic Δ40p53:TAp53 ratio (KJC40/DO-1) was significantly greater (*p* < 0.01) in metastatic samples obtained from the brain (median: 0.65, IQR: 1.11) compared to metastatic samples obtained from the skin (median: 0, IQR: 0.01). No further differences in the expression ratio of p53 isoforms compared to TAp53 were observed between different metastatic sites (Additional file 1: Fig. S3D & E).

In a subset of matched primary and metastatic samples (n = 20) no significant differences were identified in p53 isoform expression or isoform:TAp53 ratio between primary tumours and matched metastases (Additional file 1: Fig. S4A-D).

### Elevated nuclear TAp53 isoforms correlate with later stages of melanoma, while elevated nuclear Δ40p53 isoform correlates with less advanced melanoma

Next, the correlation between clinical parameters and p53 isoform expression was examined in primary melanomas (see Additional file 1: Table S1 for available data). Elevated nuclear TAp53 H-scores were found to be positively correlated with later cancer stages (Spearman’s rho: 0.448, *p* < 0.05, did not pass multiple comparison correction), while elevated nuclear Δ40p53 H-scores were negatively correlated with Clarke’s microanatomical level (Spearman’s rho: -0.323, *p* < 0.05, did not pass multiple comparison correction) (Table 1). Similarly, nuclear Δ40p53:TAp53 ratios (KJC40/DO-1) negatively correlated with disease stage (Spearman’s rho: − 0.614, *p* < 0.01) and Breslow thickness (Spearman’s rho: − 0.568, *p* < 0.0001). Nuclear p53β:TAp53 ratios (KJC8/DO-1) negatively correlated with Breslow thickness (Spearman’s rho: -0.369, *p* < 0.001) (Table 1); overall, suggesting that while elevated nuclear Δ40p53 detection and p53β:TAp53 ratio (KJC8/DO-1) are associated with better prognostic features, elevated nuclear TAp53 isoforms are associated with more aggressive melanoma, corroborating evidence that dysregulated wild-type p53 may function as an oncogene in melanoma [4, 6]. At the basal level, both Δ40p53 and p53β have been found to harbour tumour-suppressive functions, such as inducing the expression of p53 target genes involved in apoptosis and cell-cycle arrest [21, 34]. While these studies were conducted in breast cancer [21, 34], they may hint at what drives the association between Δ40p53 or p53β:TAp53 and less aggressive melanoma. In the case of elevated p53β:TAp53, the association with less aggressive melanoma may also be driven by lower TAp53 levels, which may indicate functionally active full-length p53 as opposed to overexpression of the tumour suppressor, which has been linked to cell survival and treatment resistance in melanoma [7].

**Table 1:** Spearman’s rho correlation between isoform H-scores or isoform:TAp53 ratios and clinical parameters in primary melanomas

H-scores		TAp53 (DO-1) H-score (nucleus)	TAp53 (DO-1) H-score (cytoplasm)	p53β (KJC8) H-score (nucleus)	p53β (KJC8) H-score (cytoplasm)	Δ133p53 (KJC133) H-score (nucleus)	Δ133p53 (KJC133) H-score (cytoplasm)	Δ40p53 (KJC40) H-score (nucleus)	Δ40p53 (KJC40) H-score (cytoplasm)
Age at Diagnosis	Correlation Coefficient	0.109	0.178	0.031	− 0.045	0.137	− 0.003	0.017	− 0.181
	*p*-value	0.372	0.144	0.799	0.708	0.255	0.977	0.886	0.131
Stage	Correlation Coefficient	0.448*	0.377	0.185	− 0.005	− 0.024	0.227	− 0.161	− 0.015
	*p*-value	**0.028**	0.070	0.376	0.980	0.911	0.275	0.441	0.943
Clarke's Microanatomical Level	Correlation Coefficient	0.293*	0.125	0.015	− 0.047	0.083	0.136	− 0.323*	− 0.155
	*p*-value	**0.028**	0.360	0.911	0.726	0.534	0.308	**0.013**	0.246
Tumour Thickness (cm)	Correlation Coefficient	0.121	0.030	0.069	0.103	0.203	0.193	− 0.141	− 0.028
	*p*-value	0.391	0.834	0.619	0.457	0.140	0.161	0.309	0.840

Isoform:TAp53 ratios		p53β:TAp53 (KJC8/DO-1) (nucleus)	p53β:TAp53 (KJC8/DO-1) (cytoplasm)	Δ133p53:TAp53 (KJC133/DO-1) (nucleus)	Δ133p53:TAp53 (KJC133/DO-1) (cytoplasm)	Δ40p53:TAp53 (KJC40/DO-1) (nucleus)	Δ40p53:TAp53 (KJC40/DO-1) (cytoplasm)
Age at diagnosis	Correlation Coefficient	− 0.073	− 0.277*	0.003	− 0.165	− 0.064	− 0.239
	*p*-value	0.552	**0.026**	0.980	0.193	0.605	0.058
Stage	Correlation Coefficient	− 0.398	− 0.206	− 0.323	− 0.050	− 0.614**	− 0.105
	*p*-value	0.054	0.347	0.123	0.820	**0.001**	0.632
Breslow thickness	Correlation Coefficient	− 0.369**	− 0.250	− 0.120	− 0.037	− 0.568***	− 0.255
	*p*-value	**0.005**	0.071	0.380	0.795	** < 0.0001**	0.065
Clarke's microanatomical level	Correlation Coefficient	− 0.144	− 0.021	0.166	0.125	− 0.232	− 0.113
	*p*-value	0.309	0.887	0.239	0.393	0.101	0.440

Significant correlations ins bold. *—*p* < 0.05 (did not pass multiple comparison correction); **—*p* < 0.01; ***—*p* < 0.0001

### Elevated detection of TAp53 and p53β isoforms are associated with worse prognosis

**Fig. 2:**
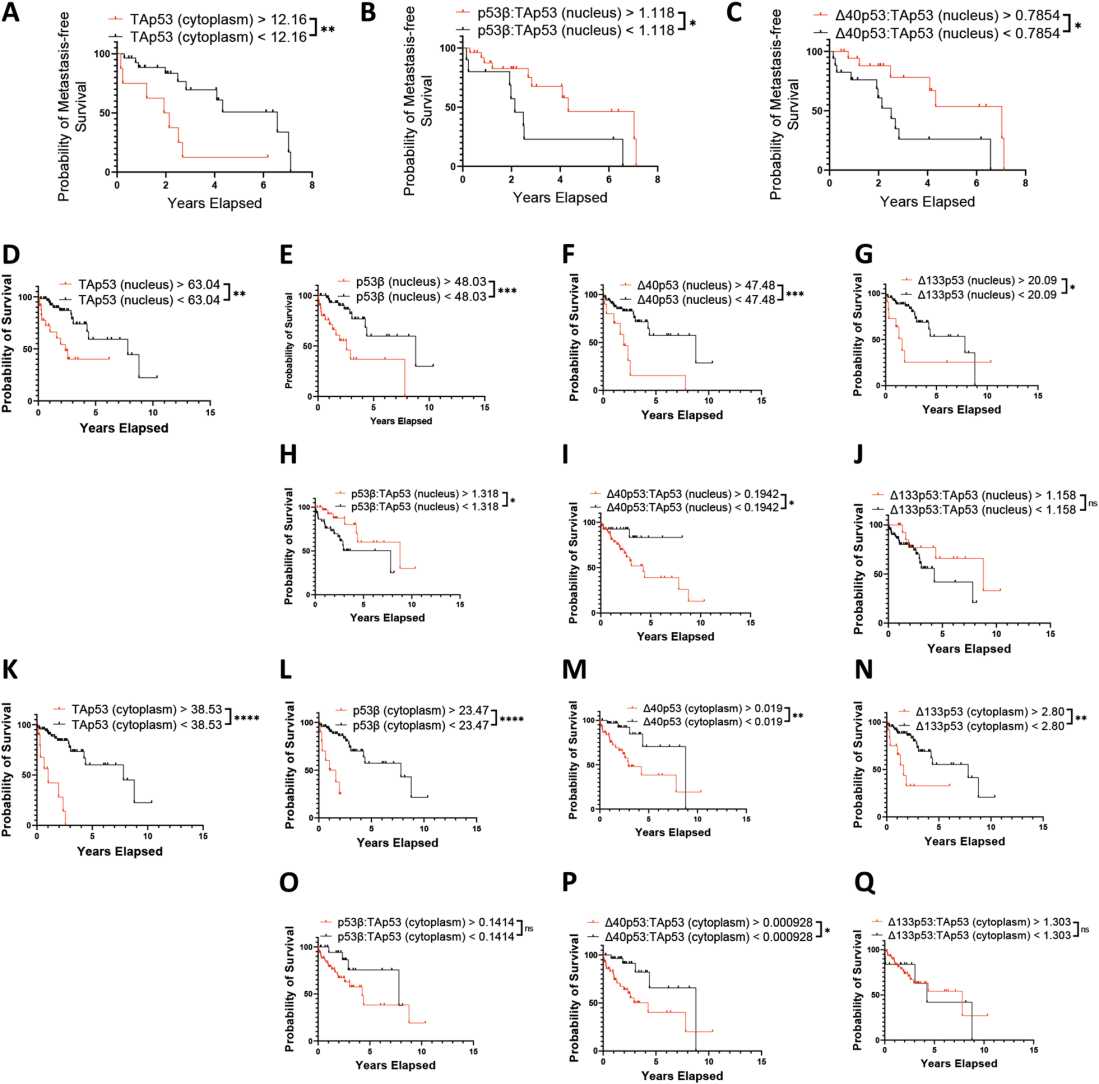
Probability of survival and metastasis based on p53 isoform expression in melanoma. Kaplan–Meier plots of metastasis in primary melanomas (n = 46) based on **A**. cytoplasmic TAp53 H-scores > 12.16 (n = 9) or < 12.16 (n = 37), **B**. relative nuclear p53β > 1.118 (n = 28) or < 1.118 (n = 19), and **C.** relative nuclear Δ40p53 > 0.7854 (n = 21) or < 0.7854 (n = 26). Kaplan–Meier plots of melanoma-specific survival in all melanomas (n = 80) based on **D**. nuclear TAp53 H-scores > 63.04 (n = 23) or < 63.04 (n = 57), **E**. nuclear p53β H-scores > 48.03 (n = 31) or < 48.03 (n = 49), **F**. nuclear Δ40p53 H-scores > 47.48 (n = 10) or < 47.48 (n = 70), **G**. nuclear Δ133p53 H-scores > 20.09 (n = 11) or < 20.09 (n = 69), **H**. relative nuclear p53β > 1.318 (n = 34) or < 1.318 (n = 46), **I**. relative Δ40p53 > 0.1942(n = 56) or < 0.1942 (n = 24), **J**. relative nuclear Δ133p53 > 1.158 (n = 16) or < 1.158 (n = 64), **K**. cytoplasmic TAp53 H-scores > 38.53 (n = 10) or < 38.53 (n = 70), **L**. cytoplasmic p53β H-scores > 23.47 (n = 10) or < 23.47 (n = 70), **M**. cytoplasmic Δ40p53 H-scores > 0.019 (n = 45) or < 0.019 (n = 35), **N.** cytoplasmic Δ133p53 H-scores > 2.80 (n = 12) or < 2.80 (n = 68), **O**. relative cytoplasmic p53β > 0.1414 (n = 51) or < 0.1414 (n = 29), **P**. relative cytoplasmic Δ40p53 > 0.0000928 (n = 43) or < 0.0000928 (n = 37), and **Q**. relative cytoplasmic Δ133p53 > 1.303 (n = 61) or < 1.303 (n = 19). Significance by Log-rank tests. ns – not significant; *—*p* < 0.05; **—*p* < 0.01; ***—*p* < 0001; ****—*p* < 0.0001. *p*-values of ≥ 0.025 did not pass multiple comparison correction (applicable to 2B and 2H)

To determine whether expression of p53 isoforms was linked to the development of metastasis or melanoma-specific survival, we determined the optimum cut-off using the Cutoff Finder application, which determined the optimal cut-off as the point with the most significant (log-rank test) split [26] and Kaplan–Meier analysis to assess the differences in metastasis development over time. Primary melanomas with cytoplasmic TAp53 (DO-1) H-scores above 12.160 (HR: 3.5; 95% CI 0.97–12.47; *p* < 0.01) were more likely to metastasize (Fig. 2A). Additionally, primary melanomas with nuclear p53β:TAp53 ratio (KJC8/DO-1) of less than 1.118 (HR: 2.7; 95% CI 0.90–8.14; *p* < 0.05, did not pass multiple comparison testing) were more likely to develop a metastasis (Fig. 2B), corroborating previous evidence that higher nuclear TAp53 expression (Table 1) and lower p53β:TAp53 ratios (KJC8/DO-1) (Table 1) are associated with more aggressive melanoma. Comparably, nuclear Δ40p53:TAp53 ratios (KJC40/DO-1) of < 0.7854 (HR: 2.9; 95% CI 1.11–7.70; *p* < 0.05) were found to be linked to a lower probability of metastasis-free survival (Fig. 2C), which is further supported by evidence that nuclear Δ40p53:TAp53 (KJC40/DO-1) ratios are negatively associated with clinical features of more aggressive melanoma (i.e., later stage and greater Breslow thickness) (Table 1). In both cases, overexpression of TAp53, might be contributing to worse prognosis driving cancer progression and treatment resistance [7].

Regarding melanoma-specific survival, nuclear DO-1 (TAp53) H-scores > 63.04 (HR: 3.4; 95% CI 1.26–9.38; *p* < 0.01, Fig. 2D), nuclear KJC8 (p53β) H -scores > 48.03 (HR: 3.8; 95% CI 1.56–9.53; *p* < 0.001, Fig. 2E), nuclear KJC40 (Δ40p53) H-scores > 47.48 (HR: 4.0; 95% CI 1.12–14.49; *p* < 0.001, Fig. 2F), and nuclear KJC133 (Δ133p53) H-scores > 20.09 (HR: 2.6; 95% CI 0.81–8.24; *p* < 0.05, Fig. 2G) are associated with worse survival outcomes in melanoma. Contrastingly, nuclear p53β:TAp53 ratios (KJC8/DO-1) > 1.318 (HR: 0.40; 95% CI 0.18–0.89; *p* < 0.05, did not pass multiple comparison testing) were associated with better survival outcomes in melanoma (Fig. 2H), while Δ40p53:TAp53 ratios (KJC40/DO-1) > 0.1942 (*p* < 0.05) continued to be associated with worse prognosis (Fig. 2I). Similar trends were observed in the cytoplasm (Fig. 2K–Q) and in primary (Fig. S5A-N) and metastatic melanoma cases (Fig. S5O-AB) independently. Elevated nuclear KJC8 scores and KJC133 scores, may be reflecting elevated Δ133p53β expression, which has not only been previously linked to worse prognosis in melanoma [35], but may be driving dedifferentiation of melanoma cells into cancer stem cell through the upregulation of pluripotency genes such as *SOX2*, *NANOG,* and *OCT3/4* [36]. Additionally, Δ133p53β may be competing with full-length p53 for DNA binding, blocking the canonical tumour-suppressor [37].

**Fig. 3:**
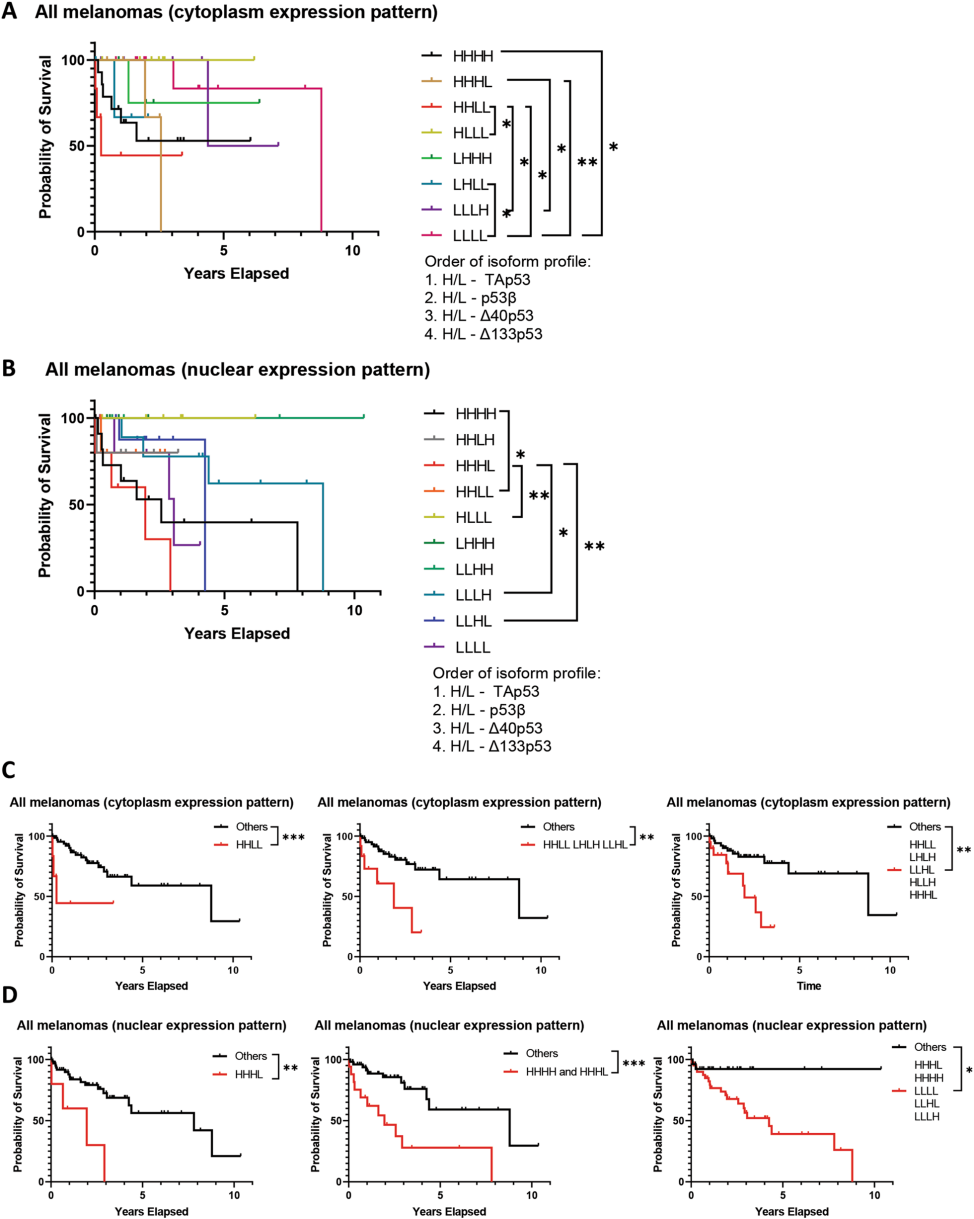
Probability of melanoma-specific survival using combined expression profiles of all p53 isoforms. Kaplan–Meier plots of melanoma-specific survival in all melanomas based on composite biomarkers of p53 isoform expression segregated into high (H) and low (L) according to the median expression of each isoform. **A**. Cytoplasmic p53 isoform expression patterns HHHH (n = 14), HHHL (n = 1; not included); HHLH (n = 5), HHLL (n = 6), HLHH (n = 2, not included), HLHL (n = 3, not included), HLLH (n = 2, not included), HLLL (n = 7), LHHH (n = 5), LHHL (n = 3, not included), LHLH (n = 2, not included), LHLL (n = 4), LLHH (n = 3, not included), LLHL (n = 6), LLLH (n = 3, not included), LLLL (n = 13). **B**. Nuclear p53 isoform expression patterns HHHH (n = 12), HHHL (n = 5), HHLH (n = 5), HHLL (n = 6), HLHH (n = 3, not included), HLHL (n = 1, not included), HLLH (n = 2, not included), HLLL (n = 6), LHHH (n = 5), LHHL (n = 2, not included), LHLH (n = 2, not included), LHLL (n = 2, not included), LLJJ (n = 4), LLHL (n = 9), LLLH (n = 9), LLLL (n = 6). **C**. Combined cytoplasmic p53 isoform expression patterns. Left: HHLL (n = 6), others (n = 67); center: HHLL, LHLH, and LLHL (n = 12), others (n = 61); right: HHLL, LHLH, LLHL, HLLH, and HHHL (n = 20), others (n = 53). **D**. Combined nuclear p53 expression patterns. Left: HHHL (n = 5), others (n = 62); center: HHHL and HHHL (n = 17), others (n = 50); right: HHHL, HHHH, LLLL, LLHL, and LLLH (n = 41), others (n = 26). Significance determined by Log-rank (Mantel-Cox) tests. *ns* not significant; *—*p* < 0.05; **—*p* < 0.01; ***—*p* < 0.001. *p*-values ≥ 0.0091 did not pass multiple comparison testing (applicable to HHHL vs LLHL, HHHL vs LLLL, HHHL vs LLLH, LLHL vs LLLH, HHHL + HHHH + LLLL + LLHL + LLLH vs all others, HHLL vs LLLL, HHLL + LHLH + LLHL + HLLH + HHHL vs all others, HHHL vs HLLL, HLLL vs LHHL, HHLL vs HLLL, HHLH vs LLHL, HHHH vs LLLL, HHLH, HLLL, LHLL vs LLLL, HHHH vs HLLL, HHLL vs LLHL, LHHL vs LLHL)

While the expression of individual p53 isoforms may be of great value from the prognostic biomarker perspective, it is the interaction between the different isoforms and full-length p53 that regulates p53 pathway activity [11, 38, 39] and may hence hold functional implications for melanoma. To investigate the complex interplay between different p53 isoforms we generated composite biomarkers of p53 isoform expression using the isoforms’ median expression to classify melanomas as having either high (H) or low (L) expression of these isoforms. To limit the possible composite biomarker classes and to not lose statistical power, we analysed nuclear and cytoplasmic expression independently and only included groups for which there were more than three melanomas. Using our novel composite biomarker, we next evaluated melanoma-specific survival between the different groups (Fig. 3). Looking at cytoplasmic expression patterns (Fig. 3A, Table 2), high expression of all isoforms (HHHH) was associated with worse survival than low expression of all isoforms (LLLL) (HR: 4.5; 95% CI 1.10–18.72; *p* < 0.05, did not pass multiple comparison correction). The best survival outcomes were attributed to elevated detection of TAp53 isoforms, accompanied by low detection of all other isoforms (HLLL), while elevated detection of TAp53 isoforms and p53β isoforms in the presence of low N-terminal isoform, Δ40p53 and Δ133p53, detection (HHLL) was associated with one of the worst prognoses. Low levels of Δ133p53 detection in the presence of high levels of all other isoforms (HHHL) was also associated with poor survival outcomes. High cytoplasmic p53β detection accompanied by low expression of all other isoforms (LHLL) was found to be associated with worse survival compared to low expression of all isoforms (LLLL) (HR: 7.5; 95% CI 0.07–804.1; *p* < 0.05, did not pass multiple comparison correction). In the nuclear expression patterns (Fig. 3B, Table 2), again elevated TAp53 in the presence of low expression of all other isoforms (HLLL) appears to be beneficial. Low nuclear detection of Δ133p53 in the presence of high detection of all other isoforms appears to be detrimental (HHHL) with the opposing profile (LLLH) showing an improved survival outcome (HR of LLLH/HHHL: 0.2; 95% CI 0.03–1.31; *p* < 0.05, did not pass multiple comparison correction). To further evaluate different combinations of the p53 isoform composite classes could be used as prognostic biomarkers, we combined the classes that presented lower median survival (Table 2) for each expression pattern (cytoplasmic or nuclear) (Fig. 3C, D). In the cytoplasmic pattern, isolated HHLL, the top three classes, and the top five classes versus all other classes predicted worst prognoses (HR: 4.6; 95% CI 0.45–47.92; *p* < 0.001, HR: 4.3; 95% CI 0.94–20.10; *p* < 0.01, and HR: 3.6; 95% CI 1.17–11.23; *p* < 0.01, respectively) (Fig. 3C). In the nuclear pattern, isolated HHHL, the top two classes, and the top five classes versus all other classes predicted worst prognoses (HR: 4.9; 95% CI 0.63–38.23; *p* < 0.01, HR: 4.0; 95% CI 1.40–11.34; *p* < 0.001, and HR: 5.2; 95% CI 2.15–12.53; *p* < 0.05, respectively, did not pass multiple comparison correction) (Fig. 3D). For the nuclear staining, the combined increased or decreased staining of DO-1 and KJC8 antibodies can predict worse outcomes, with more flexibility for the staining of antibodies that detect N-terminally truncated variants. Given that p53 functions as a transcriptional factor in the nucleus, these observations underpin important roles of p53 variants with intact transactivation domains in melanoma. Given that the composite biomarker analysis was underpowered, only limited conclusions can be drawn at this point, but the findings support larger biomarkers studies of this kind.

**Table 2:** Median survival of composite cytoplasmic or nuclear isoform expression patterns

Cytoplasmic expression pattern	n	Censored	Deaths	Median survival (years)
HHLL (DO-1^bright^, KJC8^dim/−^, KJC40^dim/−^, KJC133^dim/−^)	6	3	3	0.25
LHLH (DO-1^dim/−^, KJC8^bright^, KJC40^dim/−^, KJC133^bright^)	3	2	1	1.87
LLHL (DO-1^dim/−^, KJC8^dim/−^, KJC40^bright^, KJC133^bright^)	3	1	2	1.91
HLLH (DO-1^bright^, KJC8^dim/−^, KJC40^dim/−^, KJC133^bright^)	3	2	1	2.33
HHHL (DO-1^bright^, KJC8^bright^, KJC40^bright^, KJC133^dim/−^)	5	3	2	2.57
LLLH (DO-1^dim/−^, KJC8^dim/−^, KJC40^dim/−^, KJC133^bright^)	6	5	1	5.76
LLLL (DO-1^dim/−^, KJC8^dim/−^, KJC40^dim/−^, KJC133^dim/−^)	13	11	2	8.79
HHHH (DO-1^bright^, KJC8^bright^, KJC40^bright^, KJC133^bright^)	14	8	6	Undefined
HLLL (DO-1^bright^, KJC8^dim/−^, KJC40^dim/−^, KJC133^dim/−^)	7	7	0	Undefined
LHHH (DO-1^dim/−^, KJC8^bright^, KJC40^bright^, KJC133^bright^)	5	4	1	Undefined
LHLL (DO-1^dim/−^, KJC8^bright^, KJC40^dim/−^, KJC133^dim/−^)	4	3	1	Undefined
LLHH (DO-1^dim/−^, KJC8^dim/−^, KJC40^bright^, KJC133^bright^)	3	3	0	Undefined

Nuclear expression pattern	n	Censored	Deaths	Median survival (years)
HHHL (DO-1^bright^, KJC8^bright^, KJC40^bright^, KJC133^dim/−^)	5	1	4	1.95
HHHH (DO-1^bright^, KJC8^bright^, KJC40^bright^, KJC133^bright^)	12	5	7	2.57
LLLL (DO-1^dim/−^, KJC8^dim/−^, KJC40^dim/−^, KJC133^dim/−^)	6	3	3	3.05
LLHL (DO-1^dim/−^, KJC8^dim/−^, KJC40^bright^, KJC133^dim/−^)	9	7	2	4.25
LLLH (DO-1^dim/−^, KJC8^dim/−^, KJC40^dim/−^, KJC133^bright^)	9	5	4	8.79
HHLH (DO-1^bright^, KJC8^bright^, KJC40^dim/−^, KJC133^dim/−^)	5	4	1	Undefined
HHLL (DO-1^bright^, KJC8^bright^, KJC40^dim/−^, KJC133^dim/−^)	6	5	1	Undefined
HLLL (DO-1^bright^, KJC8^dim/−^, KJC40^dim/−^, KJC133^dim/−^)	6	6	0	Undefined
LHHH (DO-1^dim/−^, KJC8^bright^, KJC40^bright^, KJC133^bright^)	5	5	0	Undefined
LLHH (DO-1^dim/−^, KJC8^dim/−^, KJC40^bright^, KJC133^bright^)	4	4	0	Undefined

Order of isoforms in expression pattern 1. TAp53, p53β, Δ40p53, Δ133p53. Significant comparisons shown in Fig. 3A, B. *H* high, *L* low

### Correlation between p53 isoform expression and *BRAF *and *TP53* mutation status

**Fig. 4:**
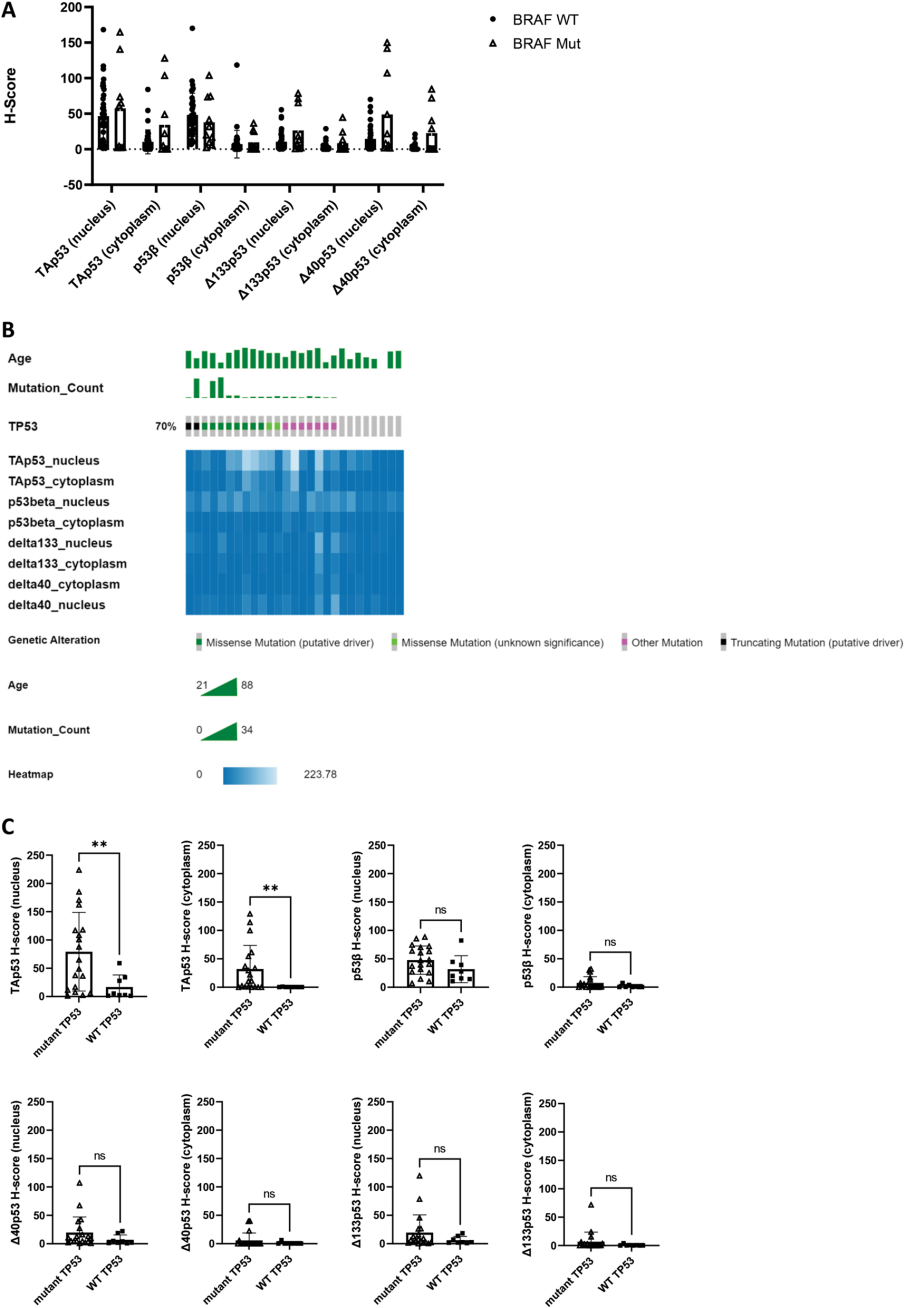
Relationship between *BRAF* and *TP53* mutation status and p53 isoform expression. **A**. p53 isoform H-scores by *BRAF* mutation status (wild-type n = 39, mutant n = 10). **B**. Oncoplot *TP53* mutations. Age, number of mutations in each sample and type of most malignant mutation shown in the top three rows followed by a heatmap of p53 isoform H-scores in sequenced samples. The plot was created with cBioportal (3). **C**. p53 isoform H-scores by *TP53* mutation status (wild-type n = 8, mutant n = 19)

*BRAF* mutations are widely described in melanoma and known to be linked to worse prognosis and chemoresistance [40]. Given that p53 and BRAF may interact in melanoma tumorigenesis and treatment response [41, 42] we decided to investigate whether there were any associations between p53 isoforms and *BRAF* mutation status. There were no differences in isolated p53 isoform detection between melanomas harbouring a *BRAF* mutation and melanomas with wild-type *BRAF* (Fig. 4A).

To determine whether *TP53* mutations may be driving p53 isoform expression, we sequenced *TP53* in 30 FFPE melanoma samples of varying TAp53 expression levels (see methods for details). Three of the amplicon libraries did not pass quality control due to insufficient yield and the remaining 27 libraries were pooled and sequenced using Illumina technology. Out of the 27 samples, all but eight harboured at least one variant (Fig. 4B), with single nucleotide variants (SNVs) being by far the most predominant class (121 SNVs, 2 multi nucleotide variants, and 2 deletions). Most variants were either found in introns (n = 47) or resulted in missense mutations (n = 45). Except for TAp53, which was found to be more highly expressed in FFPE samples with mutant *TP53*, no isoform’s expression differed between wild-type and mutant *TP53* samples (Fig. 4C). Mutations were observed in various p53 isoform composite biomarker classes; however, a higher percentage of mutated samples was observed in HHHH class, whereas wild-type *TP53* was found predominantly in classes with low TAp53 (Additional file 1: Fig. S6A). Most *TP53* variants were only present in one sample and no single variant could be linked to differences in TAp53 expression between mutant and wild-type *TP53* samples (Additional file 4: Table S4). Supporting the lack of predictive biomarker potential in melanoma [3], *TP53* mutation, was not significantly associated with melanoma-specific survival, metastasis-free survival, Breslow thickness, or Clarke’s microanatomical level (Additional file 1: Fig. S6B-E).

## Discussion

Here we demonstrated that p53 isoforms detected through IHC in FFPE melanoma samples using a suite of C- and N-terminal p53 isoform-specific antibodies (Fig. 1) harbour biomarker potential. KJC40 staining (Δ40p53) correlated with less aggressive melanoma but worse prognosis and DO-1 (TAp53) and KJC8 (p53β) staining correlated with more advanced and aggressive melanoma and worse prognosis (Table 1, Figs. 2 and 3). Composite biomarkers comprised of the expression of multiple p53 isoforms revealed a potentially complex interplay between the different isoforms and their relevance to prognosis. Such composite biomarkers highlight the need to consider p53 isoforms, not in isolation but as a connected network of redundant, synergistic, and antagonistic players [27, 43, 44]. In this context, elevated cytoplasmic DO-1 (TAp53) and KJC8 (p53β) staining in the presence of low KJC40 and KJC133 staining was associated with the worst survival outcomes (Fig. 3, Table 2).

Contrary, to our breast cancer study [12], we identified staining ranging from weak to strong across all isoforms (Fig. 1B). All isoforms were found to be more highly detected in the nucleus (Fig. 1), yet nuclear and cytoplasmic staining generally correlated (Fig. 1D). This is consistent with our previous findings in breast cancer [12] and with the isoforms exerting control over the nuclear transcriptional activity of p53 [15]. P53β isoforms did not correlate with any of the assessed N-terminal variants (TAp53, Δ40p53, or Δ133p53) in the nucleus (Additional file 1: Fig. S2A). Observed differences and similarities between our IHC study in breast cancer [12] and the findings described herein may be related to differences in *TP53* mutation status or other genetic variants within *TP53* that regulate p53 isoform expression such as mutation in the internal ribosome entry sites, introns, or in splice sites [45–48]. Additionally, with different tissue types known to express varying levels of p53 isoforms [15], the different cellular origins (melanocytes and epithelial cells) may also contribute to the different p53 isoform expression patterns of melanoma and breast cancer. Finally, other factors such as pathogenic infections, the expression of transcription and splicing factors involved in isoform expression, and the stress context of cells may determine p53 isoform expression patterns in different tissues [34, 49–53].

With KJC8 staining being the predominant nuclear staining in melanoma (Fig. 1A, B), questions arise around their potential regulation of the p53 transcription factor by p53β. Herein, we have shown that elevated nuclear KJC8 staining is associated with worse probability of survival (Fig. 2E), potentially indicating a role for the p53β isoforms in driving tumour progression, which has also been reported by others [35]. Yet increased KJC8/DO-1 ratios were associated with reduced Breslow thickness (Table 1), reduced probability of metastasis (Fig. 2B), and a better probability of survival (Fig. 2H), suggesting that it is not the p53β isoforms in isolation that are important for correlation with clinicopathological features, but their expression relative to other isoforms [27]. p53β has been previously found to enhance p53 transcriptional activity of key target genes involved in cell cycle regulation and apoptosis [17, 34], which may indicate that elevated p53β may enhance p53’s function as a tumour suppressor even when p53 expression is low (high p53β:TAp53 expression).

Nonetheless, elevated KJC8 staining may also harbour oncogenic potential underlying its association with worse survival outcomes, particularly when DO-1 staining is also elevated (Fig. 3) and p53 is potentially mutated (Fig. 4C). We hypothesise that the p53β isoforms may contribute to melanoma progression by promoting dedifferentiation. A finding from our previous study showed that primary melanoma cultures with elevated p53β expression formed non-adherent spheres, while cells from the same patient with low p53β expression grew in an adherent monolayer [17]. With sphere-forming capacity frequently used as a surrogate for a stemness phenotype [54], p53β isoforms may be involved in cancer stem cell (CSC) regulation. CSCs have previously been linked to enhanced cancer recurrence and treatment evasion, driving poorer survival outcomes [55], thus p53β may not only be a marker of worse prognosis, but may contribute to cancer aggressiveness by positively regulating dedifferentiation into CSCs. In support of this, p53β expression was associated with serous and poorly differentiated ovarian cancers and correlated with worse recurrence-free survival in patients with functional p53 [47]. Further, a recent study highlighted that Δ133p53β was associated with an increased probability of melanoma recurrence as well as a reduction in the time for the primary tumour to metastasise to the brain [35], highlighting worse prognosis linked to p53β isoforms that may guide the need to select more aggressive treatment approaches such as systemic treatments in addition to surgical resection to prolong survival [56]. Given that p53 isoform function is cell- and context-specific [27] and p53β isoforms with different N-terminal truncations are likely to have varying effects [27], it is difficult to ascertain how exactly p53β isoforms contribute to worse survival and perhaps increased cancer stem cell potential. However, we do know that isoforms may interact, shorter p53 isoforms can oligomerise with p53α and modulate its capacity to transactivate target genes [34, 53]. The exact effect p53β has likely depends on the cellular with previous studies having shown that in the absence of treatments, p53β isoforms may inhibit cell growth and induce senescence in normal human fibroblasts, T cells, and MCF-7 breast cancer cells [34, 57, 58], it may drive proliferation following treatment with TG003 (a CDK inhibitor) [34]. We hypothesise that p53β in the presence of DNA-damaging therapies, such as chemotherapeutic agents, impairs the transcriptional activity of p53α, driving the cancer cell towards survival and perhaps dedifferentiation and thus contributing to worse patient survival outcomes following treatment. Future studies should investigate whether systemic treatment of melanoma expressing high p53β is specifically associated with this worse survival and aim to determine the molecular mechanisms underpinning this potential observation.

DO-1 staining was the predominant stain in the cytoplasm of melanomas (Fig. 1A and C) and greater staining of cytoplasmic DO-1 was associated with reduced metastasis-free survival and reduced melanoma-specific survival (Fig. 2A and K). Elevated nuclear DO-1 staining was also associated with worse survival outcomes (Fig. 2D). Similarly, in a cohort of 140 benign and malignant melanocytic lesions, p53 expression, detected via IHC with the DO-7 antibody which detects a similar epitope to DO-1, was found to increase from benign nevi to malignant melanomas [59]. Further, p53 mutation was found to correlate with greater p53 expression [59], as found in this study (Fig. 4C). In these cases, p53 may have lost its function or acquired a gain-of-function mutation, enabling the mutant protein to drive cancer progression as opposed to suppressing it [60].

KJC40 staining was found to correlate with less advanced melanoma (Table 1), yet also with worse survival outcomes (Fig. 2). These findings are in line with Δ40p53 being a two-faced player in cancer [21, 31, 33]. Previous findings from our lab, have indicated that at the basal level, i.e., in the absence of chemotherapeutic agents, Δ40p53 isoforms may act similarly to p53, suppressing oncogenic traits [21]. This would support expression of these isoforms positively correlating with less advanced stages of cancer (Table 1). However, when considering the survival curves, patients are likely to have undergone various treatments for their advanced melanoma prior to succumbing to the disease. In these instances, elevated expression of Δ40p53 isoforms may have adversely affected treatment sensitivity, driving melanoma survival and growth. We have observed this in breast cancer models, where elevated Δ40p53 expression impaired p53’s canonical response to doxorubicin, promoting cancer survival, DNA repair and proliferation, while inhibiting apoptotic signalling [31]. Similarly, Δ40p53 was found to impair the upregulation of p53 target genes following cisplatin treatment of melanoma models [17]. In melanoma cell lines Δ40p53β has also been found to be elevated in vemurafenib (*BRAF* inhibitor) resistant cell lines [61], highlighting that Δ40p53 may serve as a potential biomarker to select more individualised and appropriate treatments for melanoma patients. Other studies have also reported dual roles for Δ40p53, which can act independently to p53, and both enhance or inhibit p53’s canonical functions (reviewed in [62]).

Further nuances in how isoforms may work in concert could be derived from composite biomarker analysis, where DO-1^bright^ KJC8^bright^ KJC40^dim/−^ KJC133^dim/−^ (HHLL) showed the worst survival outcomes. The complex interplay between different isoforms may also provide a potential explanation for conflicting findings between various studies hoping to uncover the function of individual p53 isoforms (reviewed in [10]). In this context, our findings in breast cancer have shown that the upregulation of Δ40p53 led to increased stability and levels of other isoforms due to heterotetramer formation and decreased proteasomal degradation [31, 33]. Thus, the composite biomarker evaluation in tissues may entail the state of activation and interaction of the isoforms. While our composite biomarker analysis lacks statistical power it is an important first step regarding p53 isoforms as an interconnected system.

Pending further validation, the isoforms may support oncologist-patient decision-making in selecting a more aggressive treatment regime (for melanomas with elevated TAp53 and p53β for example) or support the rejection of chemotherapy or *BRAF* inhibition for melanomas with elevated Δ40p53, where the isoform may drive unfavourable treatment responses [31, 33, 61]. However, in the absence of an in-depth understanding of how the different p53 isoforms contribute to potentially more aggressive cancer (TAp53 and p53β) and a lack of therapies that can directly target the various p53 isoforms, their role in melanoma needs to be considered conservatively and future studies should aim to establish the isoforms’ contribution to pathophysiology through in vitro knockout and overexpression studies, and the use of animal models. Only with such studies, would it be possible to identify the specific functions of the p53 isoforms and compensatory mechanisms among these isoforms, which may contribute to another isoform’s expression. While such in-depth molecular studies are ongoing, prognostic biomarkers, such as the putative ones identified within this manuscript still offer value to patients and oncologists, who can make informed life decisions about their prognosis.

There are several limitations of the study, including the retrospective nature of the cohort, the use of FFPE tissues of varying quality and the limited sample size. Future studies should aim to validate these findings in larger, prospective cohorts and consider the use of tissue microarrays for higher throughput or fresh melanoma and control samples, where immunofluorescence may be used as an alternative technique. Additionally, the results need to be considered in the context of possible selection bias given that the annotation areas were selected by researchers, though every effort was made to select annotations representative of the whole melanoma, to minimise such bias (see Additional file 5 for examples of selected annotations across a range of melanomas). Moreover, in the current study, we were only able to look at C- and N- terminal truncations in isolation. Given that p53 isoforms harbour C- and N- terminal truncations simultaneously [27] and, for example, different N-terminal variants of the p53β isoform are likely to have different biological functions [27], it is very hard to interpret the pathophysiological contribution of all p53β isoforms together. Future studies should aim to characterise isoforms by looking at both amino acid terminals together, though the lack of specific antibodies has limited such studies to western blotting. The fact that the p53 isoforms likely work in concert with each other further complicates the interpretation of findings and may be the main driver for so many conflicting findings about the role of different p53 isoforms in the literature [27, 63].

## Conclusions

While this study needs to be regarded as a pilot study due to the lack of statistical power, the use of a retrospective cohort, and the inability to query C- and N-terminal truncations together, it highlights how p53 isoform IHC analysis may provide a source of prognostic biomarkers that have been thus far lacking in melanoma and might, once fully validated, aid the decision-making process for treating oncologists and melanoma patients.

### Supplementary Information

**Additional file 1: Table S1**: Melanoma cohort characteristics. **Figure S1**: Expression of p53 isoforms in metastatic samples by metastatic site. A. Nuclear and B. cytoplasmic expression of p53 isoforms in metastatic melanoma samples obtained from lymph nodes (n = 13), the skin (n = 21), the brain (n = 8), the lungs (n = 3) and the gastrointestinal tract (incl. the mouth (n = 5). Two metastatic samples, one from the liver and one from the abdomen, were not included. Significance was determined by Kruskal-Wallis test corrected for multiple comparisons with Dunn’s multiple comparisons test. GIT – gastrointestinal tract; * - *p* < 0.05. **Figure S2**: Correlation between the expression of different p53 isoforms in the nucleus and cytoplasm. A. Correlations between p53β and other p53 isoform H-scores, B. correlations between Δ40p53 and other N-terminal p53 isoform H-scores, and C. correlation between Δ133p53 and TAp53 H-scores in the nucleus (left) and cytoplasm (right). R2 of the linear regressions and associated p-values are shown under each graph. **Figure S3**: Relative expression of truncated p53 isoforms. A. Relative expression of p53β, Δ40p53, and Δ133p53 to TAp53 of all melanomas in the nucleus (n = 119) and cytoplasm (n = 110). Relative B. nuclear and C. cytoplasmic expression of truncated p53 isoforms to TAp53 in primary (nucleus n = 69; cytoplasm n = 64) and metastatic melanomas (nucleus n = 50, cytoplasm n = 46). D. Relative nuclear and E. cytoplasmic expression of p53 isoforms in metastatic melanoma samples obtained from lymph nodes (nucleus n = 12, cytoplasm n = 11), the skin (nucleus n = 20, cytoplasm n = 18), the brain (nucleus n= 8, cytoplasm n = 8), the lungs (nucleus n = 3, cytoplasm n = 2) and the gastrointestinal tract (incl. the mouth (nucleus n = 3, cytoplasm n = 5). Two metastatic samples, one from the liver and one from the abdomen, are not included. Significance was determined through Friedman test (matched samples A) or Kruskal-Wallis test (unmatched samples B-E), corrected for multiple comparisons with Dunn’s multiple comparisons test. GIT – gastrointestinal tract; * - *p* < 0.05;** - *p* < 0.01; *** - *p* < 0.001; **** - *p* < 0.0001. **Figure S4**: p53 isoform expression in matched primary and metastatic melanomas. A. Nuclear and B. cytoplasmic p53 isoform H-scores in matched primary and metastatic melanoma samples (n = 21). C. Relative nuclear and D. cytoplasmic p53 isoform expression in matched primary and metastatic melanoma samples (n = 17). p-values of comparisons nearing statistical significance are shown. **Figure S5**: Probability of survival based on p53 isoform expression in primary and metastatic melanoma. Kaplan-Meier plots of probability of melanoma-specific survival in primary melanomas (n = 59) based on A. nuclear TAp53 H-scores > 63.04 (n = 16) or < 63.04 (n = 42), B. nuclear p53β H-scores > 60.57 (n = 19) or < 60.57 (n = 40), C. nuclear Δ40p53 H-scores > 44.30 (n = 10) or < 44.30 (n = 49), D. nuclear Δ133p53 H-scores > 17.03 (n = 10) or < 17.03 (n = 49), E. relative nuclear p53β expression >1.318 (n =26) or < 1.318 (n = 32), F. relative nuclear Δ40p53 > 0.2325(n =40) or < 0.2325 (n = 18), G. relative nuclear Δ133p53 expression >1.252 (n = 10) or < 1.252 (n = 48), H. cytoplasmic TAp53 H-scores > 20.08 (n = 12) or < 20.08 (n = 46), I. cytoplasmic p53β H-scores > 18.84 (n = 10) or < 18.84 (n = 49), J. cytoplasmic Δ40p53 H-scores > 5.252 (n = 11) or < 5.252 (n = 48), K. cytoplasmic Δ133p53 H-scores > 0.535 (n = 16) or < 0.535 (n = 43), L. relative cytoplasmic p53β expression > 0.0425 (n = 42) or < 0.0425 (n = 13), M. relative cytoplasmic Δ40p53 expression > 0.001 (n = 34) or < 0.001 (n = 21), N. relative cytoplasmic Δ133p53 > 1.303 (n = 46) or < 1.303 (n = 9), and in metastatic melanomas (n = 42) based on O. nuclear TAp53 H-scores > 66.63 (n = 13) or < 66.63 (n = 28), P. nuclear p53β H-scores > 50.35 (n = 18) or < 50.35 (n = 24), Q. nuclear Δ40p53 H-scores > 12.49 (n = 18) or < 12.49 (n = 24), R. nuclear Δ133p53 H-scores > 18.70 (n = 10) or < 18.70 (n = 32), S. relative nuclear p53β expression > 0.6186 (n = 30) or < 0.6186 (n = 11), T. relative nuclear Δ40p53 > 0.6444 (n =16) or < 0.6444 (n = 25), U. relative nuclear Δ133p53 expression > 0.2982 (n = 20) or < 0.2989 (n = 21), V. cytoplasmic TAp53 H-scores > 6.488 (n = 16) or < 6.488 (n = 25), W. cytoplasmic p53β H-scores > 1.043 (n = 20) or < 1.043 (n = 22), X. cytoplasmic Δ40p53 H-scores > 0.0265 (n = 26) or < 0.0265 (n = 16), Y. cytoplasmic Δ133p53 H-scores > 0.591 (n = 17) or < 0.591 (n = 25), Z. relative cytoplasmic p53β expression > 0.2384 (n = 24) or < 0.2384 (n = 14), AA. relative cytoplasmic Δ40p53 expression > 0.3022 (n = 12) or < 0.3022 (n = 26), AB. relative cytoplasmic Δ133p53 > 0.2548 (n = 16) or < 0.2548 (n = 22). Significance was determined by Log-rank (Mantel-Cox) tests. ns – not significant; * - *p* < 0.05; ** - *p* < 0.01; *** - *p* < 0001; **** - *p* < 0.0001. **Figure S6**: Association of *TP53* mutation status with clinical parameters. A. Percentage of melanoma cases with wild-type (n = 8) or mutated (n = 19) *TP53* divided according to the p53 isoform composite biomarker classes. B. Kaplan-Meier plots of probability of melanoma-specific survival in primary melanomas (n = 27) based on *TP53* mutation status. C. Kaplan-Meier plots of probability of metastasis-free survival in primary melanomas (n = 27) based on *TP53* mutation status. D. Breslow thickness and E. Clarke’s microanatomical level in samples with mutant and wild-type *TP53*. In A and B Significance was determined by Log-rank (Mantel-Cox) tests. *p*-values as indicated. In C and D significance was determined by Kruskal-Wallis test. ns – not significant; mut – mutant; WT – wild-type.

**Additional file 2: Table S2**. AmpliSeq Custom DNA Panel Details.

**Additional file 3: Table S3**. Illumina AmpliSeq Amplicons.

**Additional file 4: Table S4**. *TP53* Variants.

**Additional file 5:** Representative annotations of immunostained slides of melanoma specimens.

## Data Availability

The data presented in this study are available on request from the corresponding author.
