# Supplementary material for: It is not all about the alpha: elevated expression of p53β variants is associated with lower probability of survival in a retrospective melanoma cohort
Source: Cancer Cell Int. 2023 Oct 4;23:228. doi: 10.1186/s12935-023-03083-6 (PMC10548590; doi:10.1186/s12935-023-03083-6)
Supplement: Supplementary file 1 — Additional file 1: Table S1: Melanoma cohort characteristics. Figure S1: Expression of p53 isoforms in metastatic samples by metastatic site. A. Nuclear and B. cytoplasmic expression of p53 isoforms in metastatic melanoma samples obtained from lymph nodes (n = 13), the skin (n = 21), the brain (n = 8), the lungs (n = 3) and the gastrointestinal tract (incl. the mouth (n = 5). Two metastatic samples, one from the liver and one from the abdomen, were not included. Significance was determined by Kruskal-Wallis test corrected for multiple comparisons with Dunn’s multiple comparisons test. GIT – gastrointestinal tract; * - p < 0.05. Figure S2: Correlation between the expression of different p53 isoforms in the nucleus and cytoplasm. A. Correlations between p53β and other p53 isoform H-scores, B. correlations between Δ40p53 and other N-terminal p53 isoform H-scores, and C. correlation between Δ133p53 and TAp53 H-scores in the nucleus (left) and cytoplasm (right). R2 of the linear regressions and associated p-values are shown under each graph. Figure S3: Relative expression of truncated p53 isoforms. A. Relative expression of p53β, Δ40p53, and Δ133p53 to TAp53 of all melanomas in the nucleus (n = 119) and cytoplasm (n = 110). Relative B. nuclear and C. cytoplasmic expression of truncated p53 isoforms to TAp53 in primary (nucleus n = 69; cytoplasm n = 64) and metastatic melanomas (nucleus n = 50, cytoplasm n = 46). D. Relative nuclear and E. cytoplasmic expression of p53 isoforms in metastatic melanoma samples obtained from lymph nodes (nucleus n = 12, cytoplasm n = 11), the skin (nucleus n = 20, cytoplasm n = 18), the brain (nucleus n= 8, cytoplasm n = 8), the lungs (nucleus n = 3, cytoplasm n = 2) and the gastrointestinal tract (incl. the mouth (nucleus n = 3, cytoplasm n = 5). Two metastatic samples, one from the liver and one from the abdomen, are not included. Significance was determined through Friedman test (matched samples A) or Kruskal-Wallis test (unmatched sample [file 12935_2023_3083_MOESM1_ESM.docx]

**Additional file**

**Additional file 1: Table S1: Melanoma cohort characteristics.**

|  | | **Total** | **Primary** | **Mets** | ***p*-value** |
| --- | --- | --- | --- | --- | --- |
| **n (% total)** | | **123** | **71 (57.72%)** | **52 (42.28%)** |  |
| **Age (years)** | Median | 71 | 71 | 68.5 | 0.375 |
|  | Range | 21-98 | 21-98 | 28-88 |  |
| **Sex** | F (%) | 42 (34.15%) | 23 (32.39%) | 19 (36.54 %) | 0.702 |
|  | M (%) | 81 (65.59%) | 48 (67.61%) | 33 (63.46 %) |  |
| **Stage** | 1 (%) | 3 (2.44%) | 3 (4.23%) | 0 | N/A |
|  | 2 (%) | 2 (1.63%) | 2 (2.82%) | 0 |  |
|  | 3 (%) | 13 (10.57%) | 9 (12.68%) | 4 (7.69%) |  |
|  | 4 (%) | 11 (8.94%) | 11 (15.49%) | 0 |  |
|  | Missing (%) | 94 (76.42%) | 46 (64.79%) | 48 (92.31%) |  |
| ***BRAF* Mutation** | WT (%) | 39 (31.71%) | 25 (35.21%) | 14 (26.92%) | 0.464 |
|  | Mutant (%) | 10 (8.13%) | 8 (11.27%) | 2 (3.85%) |  |
|  | Missing (%) | 74 (60.16%) | 38 (53.52%) | 36 (69.23%) |  |
| **Breslow Thickness (mm)** | Median |  | 6.23 |  |  |
|  | Range |  | 0.4-65 |  |  |
|  | Missing |  | 13 (18.31%) |  |  |
| **Clarke's Microanatomical Level** | 1 |  | 1 (1.41%) |  |  |
|  | 2 |  | 2 (2.82%) |  |  |
|  | 3 |  | 10 (14.08%) |  |  |
|  | 4 |  | 24 (33.80%) |  |  |
|  | 5 |  | 17 (23.94%) |  |  |
|  | Missing |  | 17 (23.94%) |  |  |
| **Development of Metastasis** | Yes |  | 28 (39.44%) |  |  |
|  | No |  | 19 (26.76%) |  |  |
|  | Unknown |  | 24 (33.80%) |  |  |

*p*-values (primary vs mets): Age – Whitney U Test; Sex & *BRAF* mutation – Fisher’s exact test. Mets: metastasis; *BRAF*: B-Raf Proto-Oncogene; WT – wild-type.

**
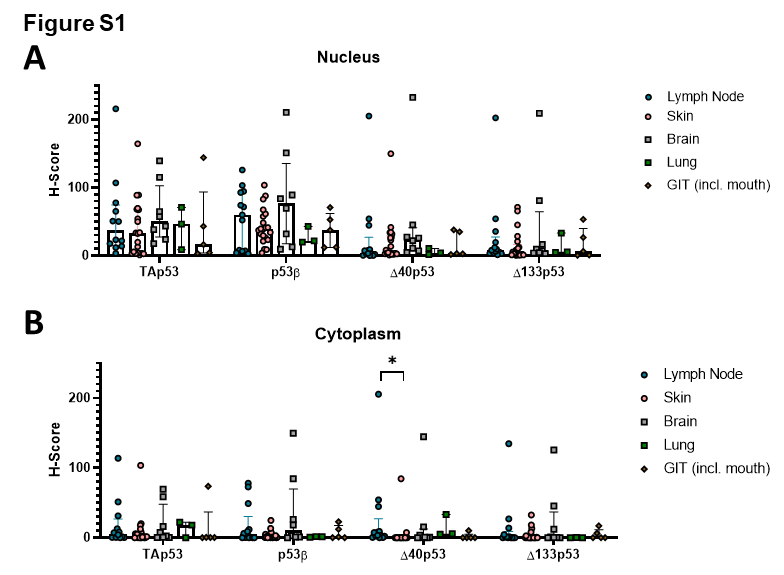
**

**Additional file 1: Figure S1: Expression of p53 isoforms in metastatic samples by metastatic site. A.** Nuclear and **B.** cytoplasmic expression of p53 isoforms in metastatic melanoma samples obtained from lymph nodes (n = 13), the skin (n = 21), the brain (n = 8), the lungs (n = 3) and the gastrointestinal tract (incl. the mouth (n = 5). Two metastatic samples, one from the liver and one from the abdomen, were not included. Significance was determined by Kruskal-Wallis test corrected for multiple comparisons with Dunn’s multiple comparisons test. GIT – gastrointestinal tract; * - *p* < 0.05.


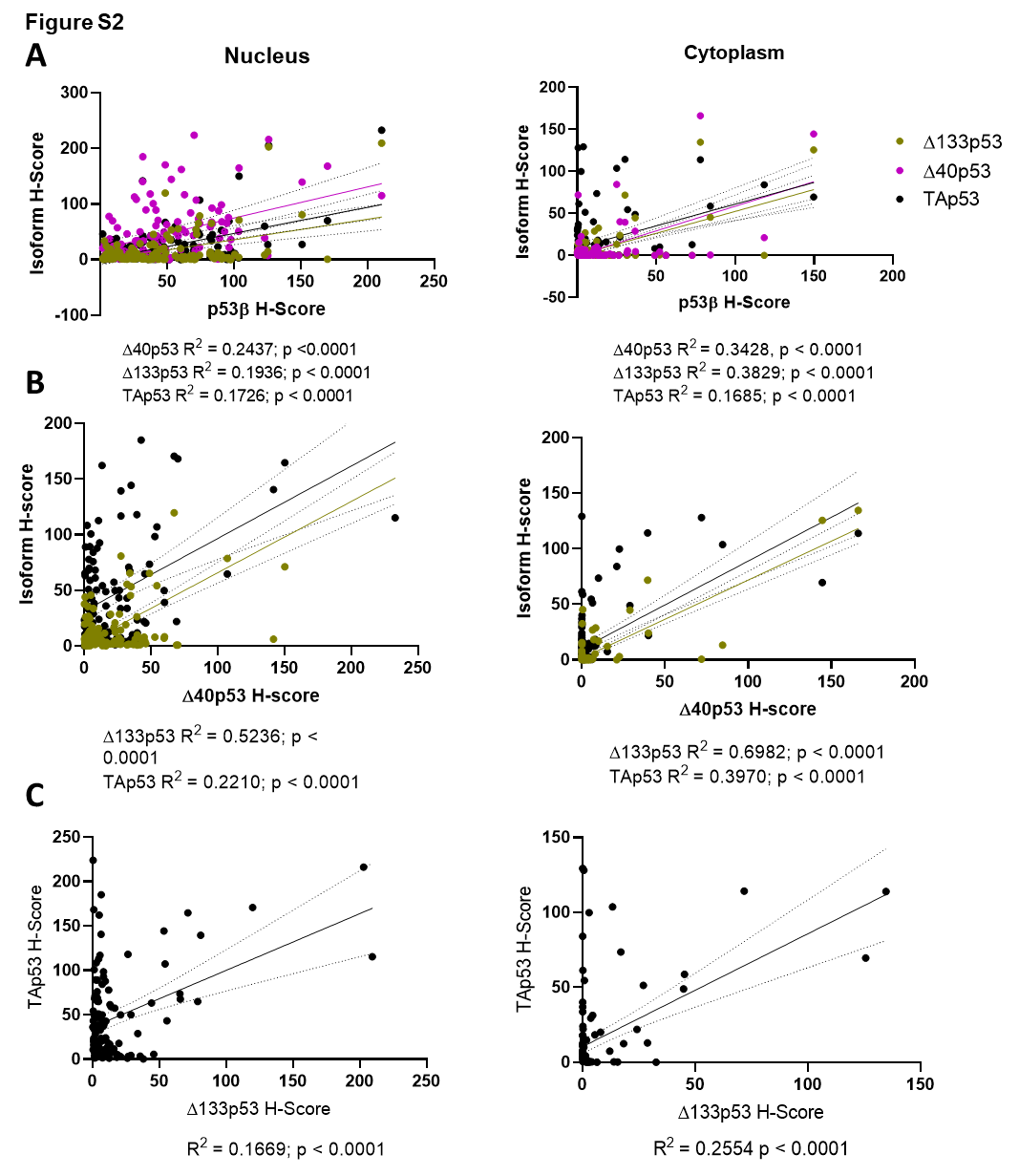


**Additional file 1: Figure S2: Correlation between the expression of different p53 isoforms in the nucleus and cytoplasm. A**. Correlations between p53β and other p53 isoform H-scores, **B.** correlations between Δ40p53 and other N-terminal p53 isoform H-scores, and **C.** correlation between Δ133p53 and TAp53 H-scores in the nucleus (left) and cytoplasm (right). R^2^ of the linear regressions and associated *p*-values are shown under each graph.


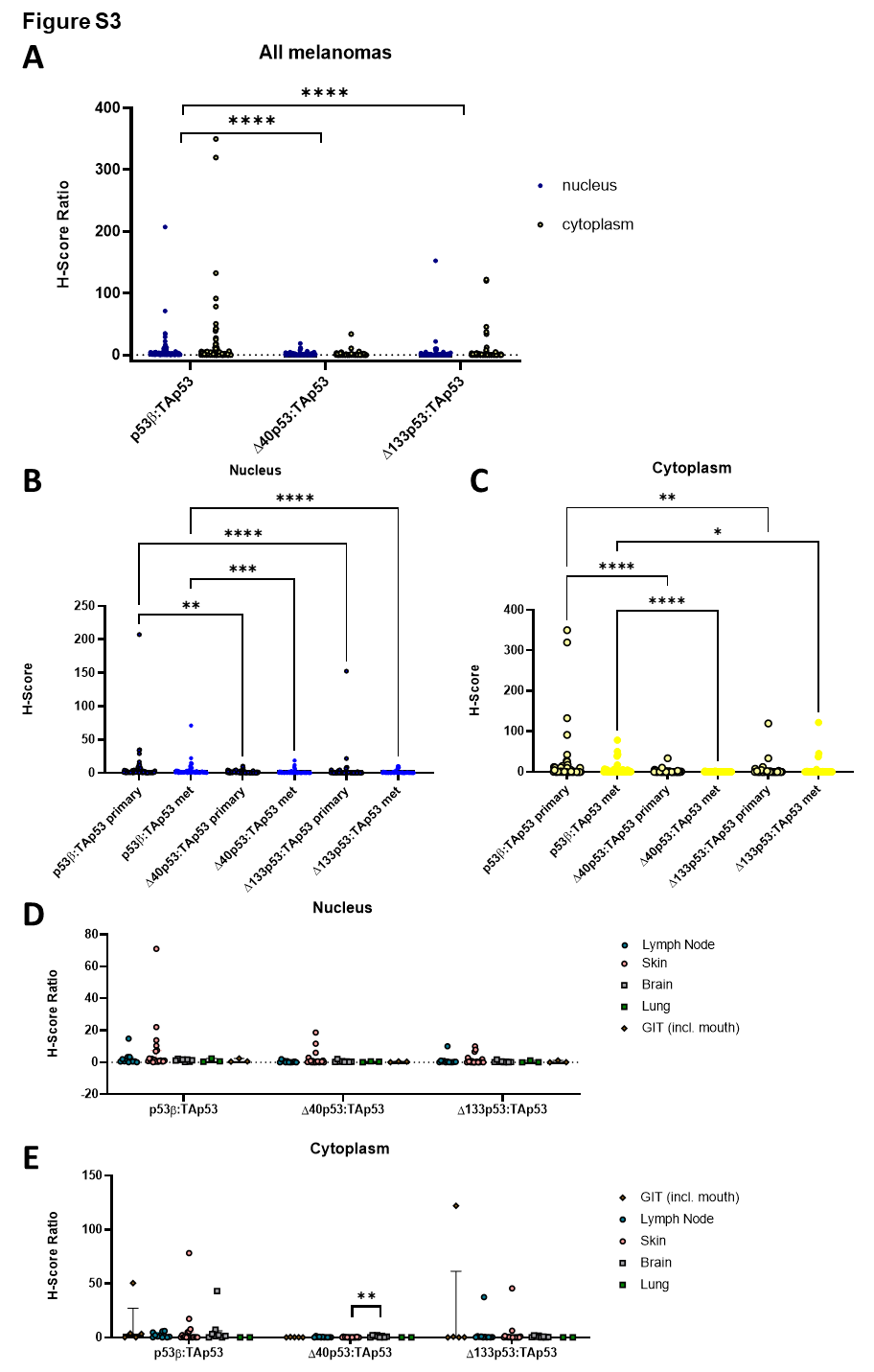


**Additional file 1: Figure S3: Relative expression of truncated p53 isoforms. A.** Relative expression of p53β, Δ40p53, and Δ133p53 to TAp53 of all melanomas in the nucleus (n = 119) and cytoplasm (n = 110). Relative **B.** nuclear and **C.** cytoplasmic expression of truncated p53 isoforms to TAp53 in primary (nucleus n = 69; cytoplasm n = 64) and metastatic melanomas (nucleus n = 50, cytoplasm n = 46). **D.** Relative nuclear and **E.** cytoplasmic expression of p53 isoforms in metastatic melanoma samples obtained from lymph nodes (nucleus n = 12, cytoplasm n = 11), the skin (nucleus n = 20, cytoplasm n = 18), the brain (nucleus n= 8, cytoplasm n = 8), the lungs (nucleus n = 3, cytoplasm n = 2) and the gastrointestinal tract (incl. the mouth (nucleus n = 3, cytoplasm n = 5). Two metastatic samples, one from the liver and one from the abdomen, are not included. Significance was determined through Friedman test (matched samples **A**) or Kruskal-Wallis test (unmatched samples **B-E**), corrected for multiple comparisons with Dunn’s multiple comparisons test. GIT – gastrointestinal tract; * - *p* < 0.05;** - *p* < 0.01; *** - *p* <0.001; **** - *p* <0.0001.


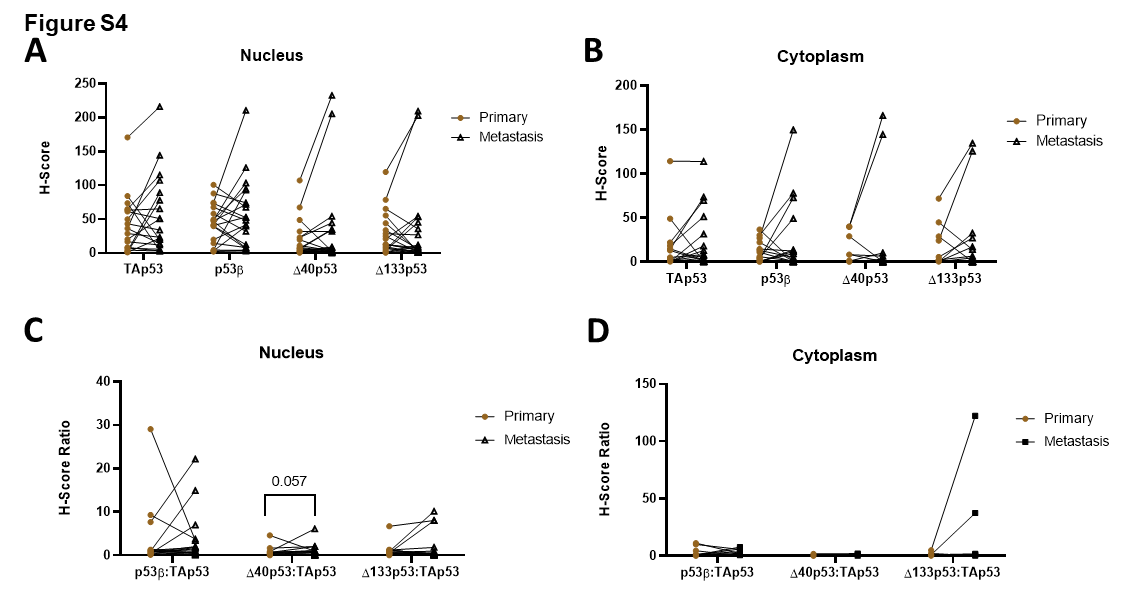


**Additional file 1: Figure S4: p53 isoform expression in matched primary and metastatic melanomas.** **A.** Nuclear and **B.** cytoplasmic p53 isoform H-scores in matched primary and metastatic melanoma samples (n=21). **C.** Relative nuclear and **D.** cytoplasmic p53 isoform expression in matched primary and metastatic melanoma samples (n=17). *p*-values of comparisons nearing statistical significance are shown.


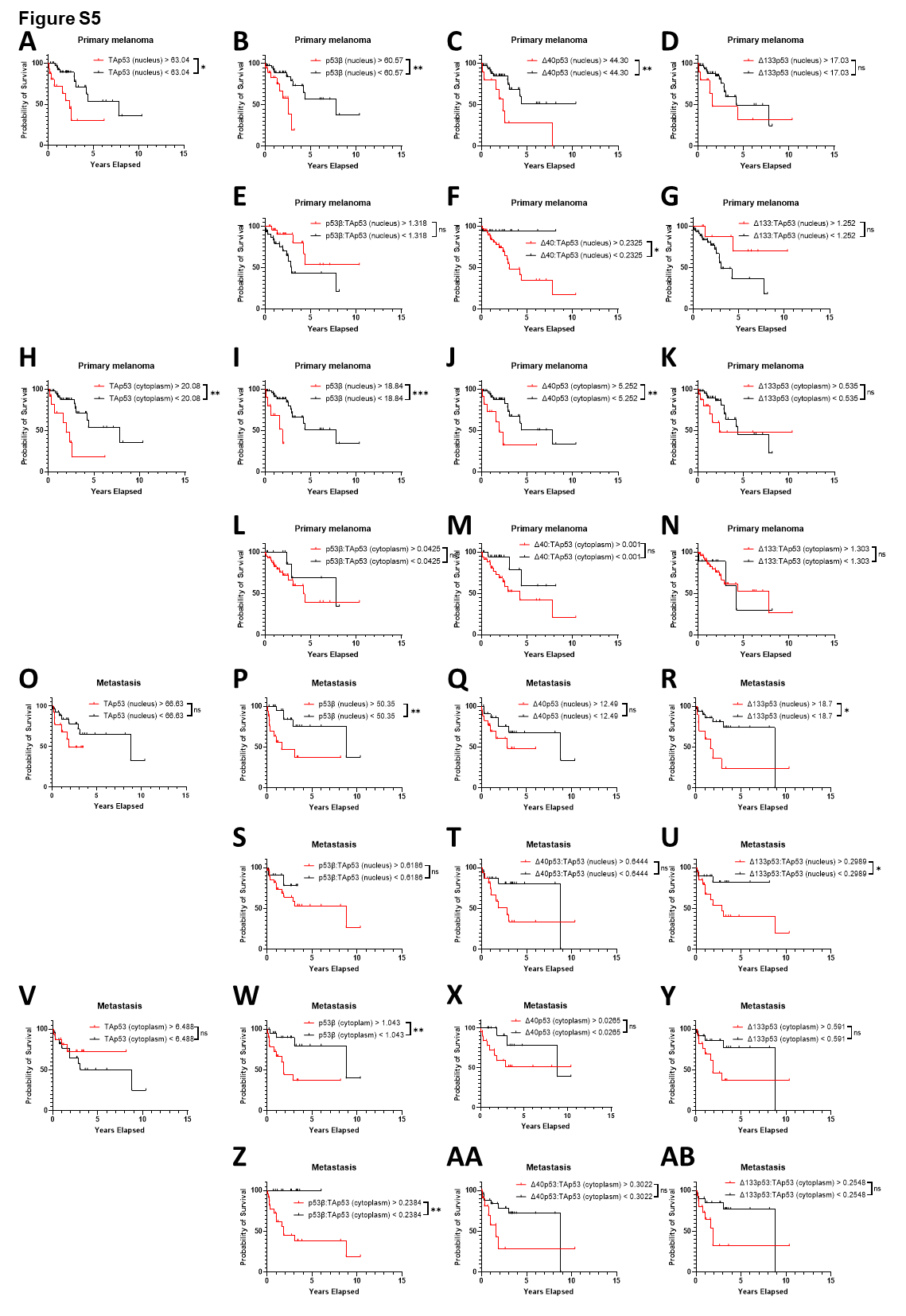


**Additional file 1: Figure S5: Probability of survival based on p53 isoform expression in primary and metastatic melanoma.** Kaplan-Meier plots of probability of melanoma-specific survival in primary melanomas (n = 59) based on **A.** nuclear TAp53 H-scores > 63.04 (n = 16) or < 63.04 (n = 42), **B.** nuclear p53β H-scores > 60.57 (n = 19) or < 60.57 (n = 40), **C.** nuclear Δ40p53 H-scores > 44.30 (n = 10) or < 44.30 (n = 49), **D.** nuclear Δ133p53 H-scores > 17.03 (n = 10) or < 17.03 (n = 49), **E.** relative nuclear p53β expression >1.318 (n =26) or < 1.318 (n = 32), **F.** relative nuclear Δ40p53 > 0.2325(n =40) or < 0.2325 (n = 18), **G.** relative nuclear Δ133p53 expression >1.252 (n = 10) or < 1.252 (n = 48), **H.** cytoplasmic TAp53 H-scores > 20.08 (n = 12) or < 20.08 (n = 46), **I.** cytoplasmic p53β H-scores > 18.84 (n = 10) or < 18.84 (n = 49), **J.** cytoplasmic Δ40p53 H-scores > 5.252 (n = 11) or < 5.252 (n = 48), **K.** cytoplasmic Δ133p53 H-scores > 0.535 (n = 16) or < 0.535 (n = 43), **L.** relative cytoplasmic p53β expression > 0.0425 (n = 42) or < 0.0425 (n = 13), **M.** relative cytoplasmic Δ40p53 expression > 0.001 (n = 34) or < 0.001 (n = 21), **N.** relative cytoplasmic Δ133p53 > 1.303 (n = 46) or < 1.303 (n = 9), and in metastatic melanomas (n = 42) based on **O.** nuclear TAp53 H-scores > 66.63 (n = 13) or < 66.63 (n = 28), **P.** nuclear p53β H-scores > 50.35 (n = 18) or < 50.35 (n = 24), **Q.** nuclear Δ40p53 H-scores > 12.49 (n = 18) or < 12.49 (n = 24), **R.** nuclear Δ133p53 H-scores > 18.70 (n = 10) or < 18.70 (n = 32), **S.** relative nuclear p53β expression > 0.6186 (n = 30) or < 0.6186 (n = 11), **T.** relative nuclear Δ40p53 > 0.6444 (n =16) or < 0.6444 (n = 25), **U.** relative nuclear Δ133p53 expression > 0.2982 (n = 20) or < 0.2989 (n = 21), **V.** cytoplasmic TAp53 H-scores > 6.488 (n = 16) or < 6.488 (n = 25), **W.** cytoplasmic p53β H-scores > 1.043 (n = 20) or < 1.043 (n = 22), **X.** cytoplasmic Δ40p53 H-scores > 0.0265 (n = 26) or < 0.0265 (n = 16), **Y.** cytoplasmic Δ133p53 H-scores > 0.591 (n = 17) or < 0.591 (n = 25), **Z.** relative cytoplasmic p53β expression > 0.2384 (n = 24) or < 0.2384 (n = 14), **AA.** relative cytoplasmic Δ40p53 expression > 0.3022 (n = 12) or < 0.3022 (n = 26), **AB.** relative cytoplasmic Δ133p53 > 0.2548 (n = 16) or < 0.2548 (n = 22). Significance was determined by Log-rank (Mantel-Cox) tests. ns – not significant; * - *p* < 0.05; ** - *p* < 0.01; *** - *p* < 0001; **** - *p* < 0.0001.


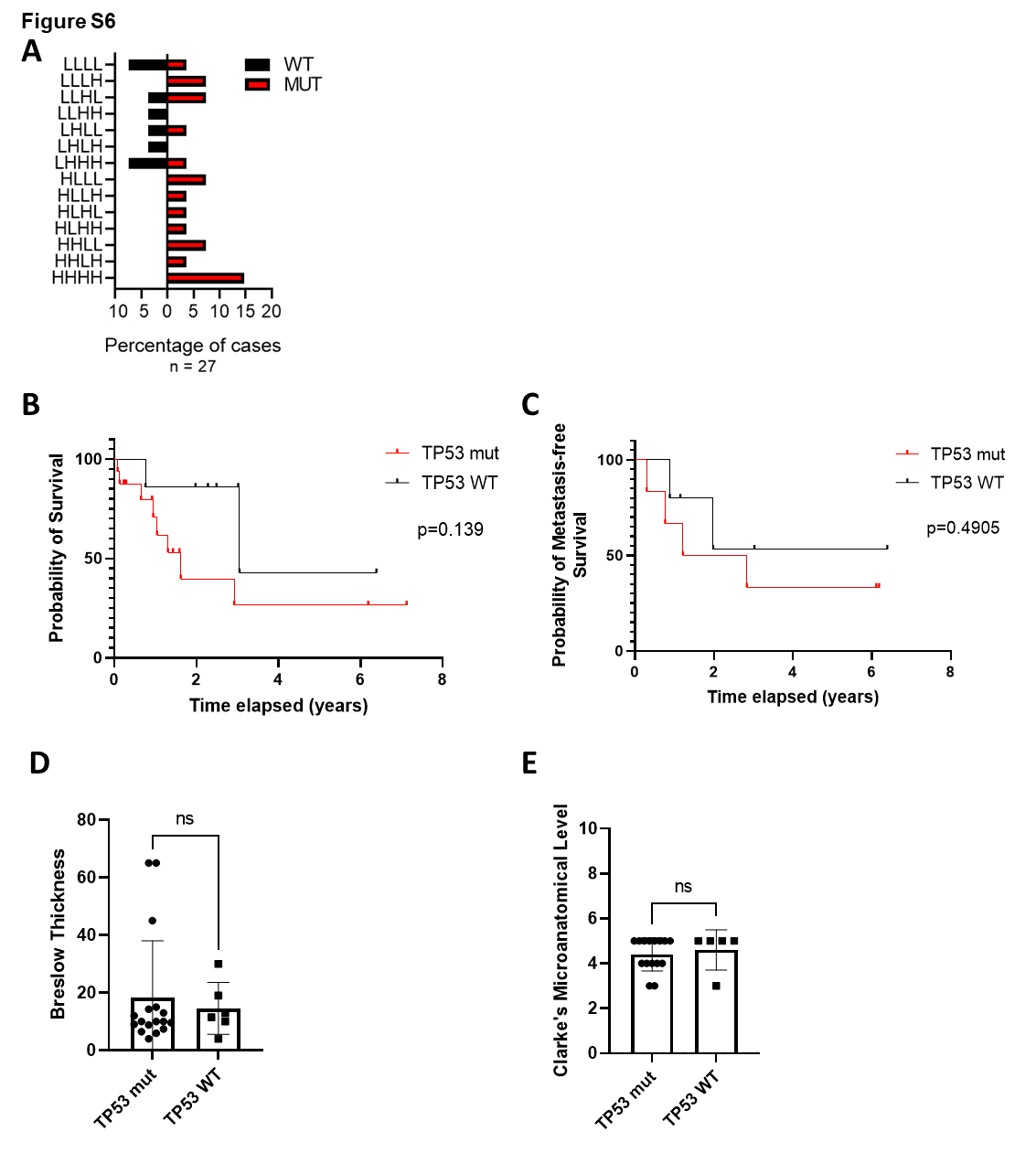


**Additional file 1: Figure S6: Association of *TP53* mutation status with clinical parameters. A.** Percentage of melanoma cases with wild-type (n = 8) or mutated (n = 19) *TP53* divided according to the p53 isoform composite biomarker classes**. B.** Kaplan-Meier plots of probability of melanoma-specific survival in primary melanomas (n = 27) based on *TP53* mutation status. **C.** Kaplan-Meier plots of probability of metastasis-free survival in primary melanomas (n = 27) based on *TP53* mutation status.  **D.** Breslow thickness and **E.** Clarke’s microanatomical level in samples with mutant and wild-type *TP53.* In A and B Significance was determined by Log-rank (Mantel-Cox) tests. *p*-values as indicated. In C and D significance was determined by Kruskal-Wallis test. ns – not significant; mut – mutant; WT – wild-type.
