# Supplementary material for: It is not all about the alpha: elevated expression of p53β variants is associated with lower probability of survival in a retrospective melanoma cohort
Source: Cancer Cell Int. 2023 Oct 4;23:228. doi: 10.1186/s12935-023-03083-6 (PMC10548590; doi:10.1186/s12935-023-03083-6)

Q2020-064-S066
Primary melanoma
Slide: 126445, TAp53

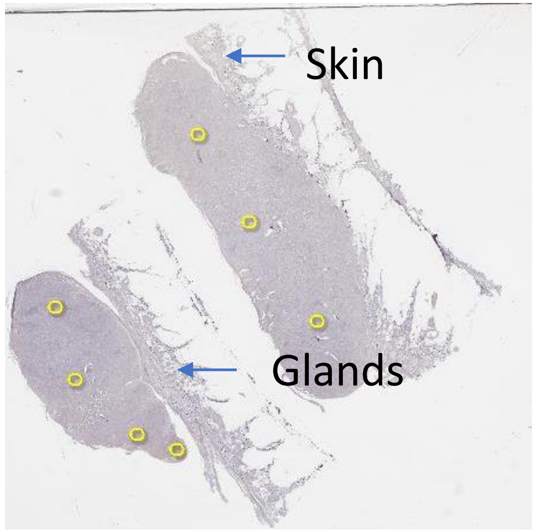

TAp53 cytoplasm low
TAp53 nucleus low
Δ40p53 cytoplasm low
Δ40p53 nucleus low

TAp53 H-scores:	p53β H-scores:	Δ133p53 H-scores:	Δ40p53 H-scores:
Cytoplasm: 0.00	Cytoplasm: 51.62	Cytoplasm: 1.67	Cytoplasm: 0.00
Nucleus: 0.25	Nucleus: 16.21	Nucleus: 38.00	Nucleus: 0.10

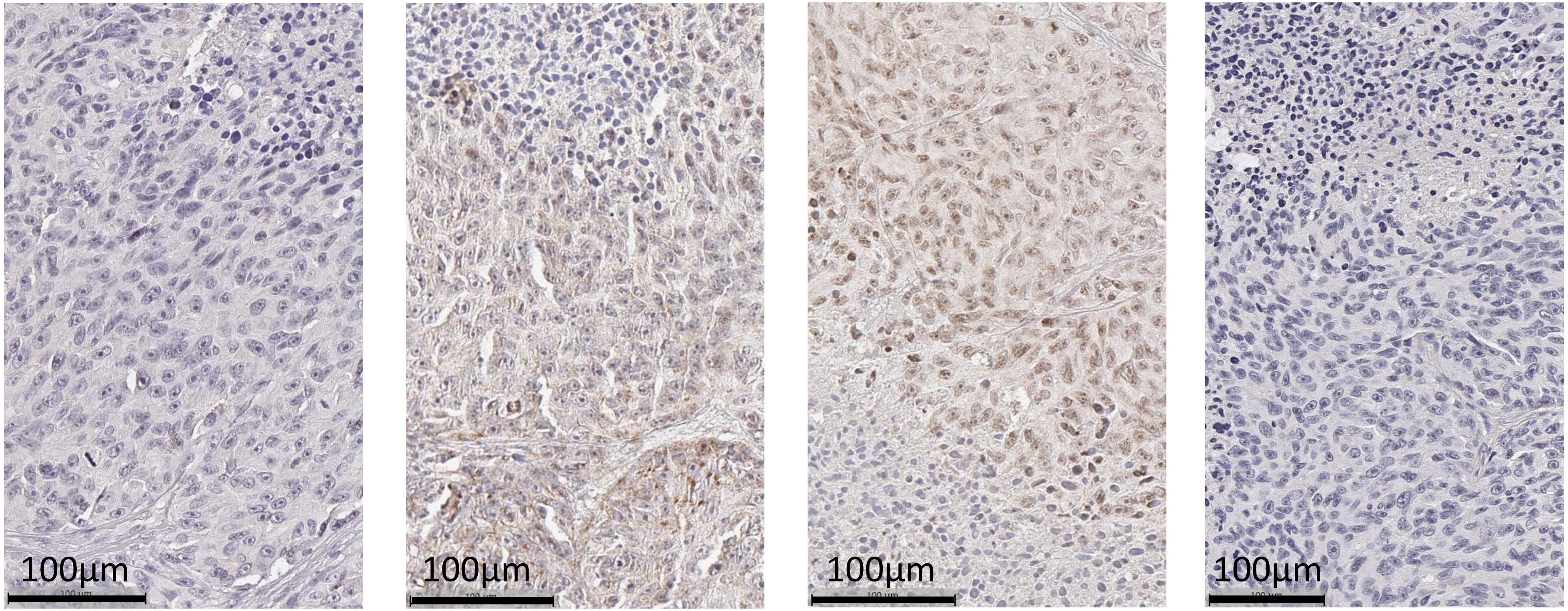

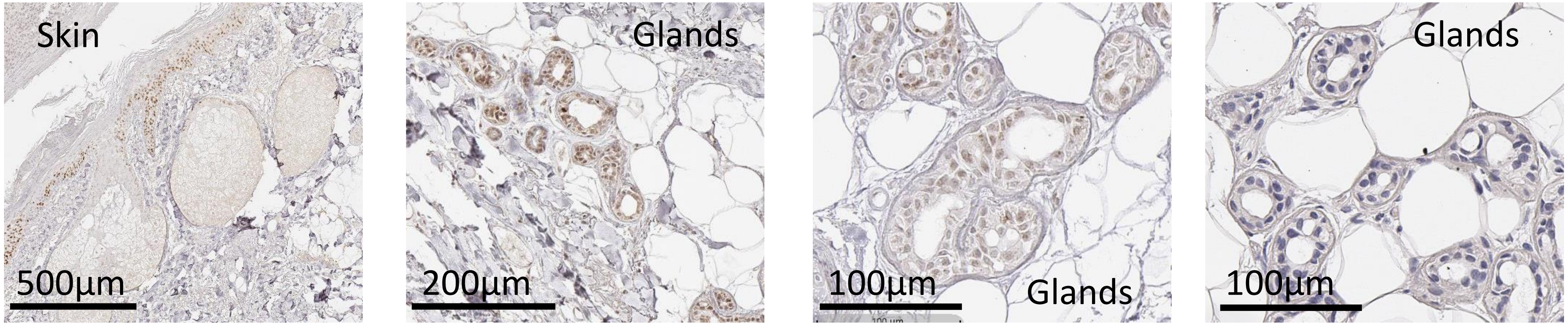

Q2020-064-S006
Primary melanoma
Slide: 126413, TAp53

TAp53 H-scores:
Cytoplasm: 128.07
Nucleus: 140.59

p53β H-scores:
Cytoplasm: 0.52
Nucleus: 31.55

Δ133p53 H-scores:
Cytoplasm: 0.57
Nucleus: 6.33

Δ40p53 H-scores:
Cytoplasm: 71.91
Nucleus: 141.70

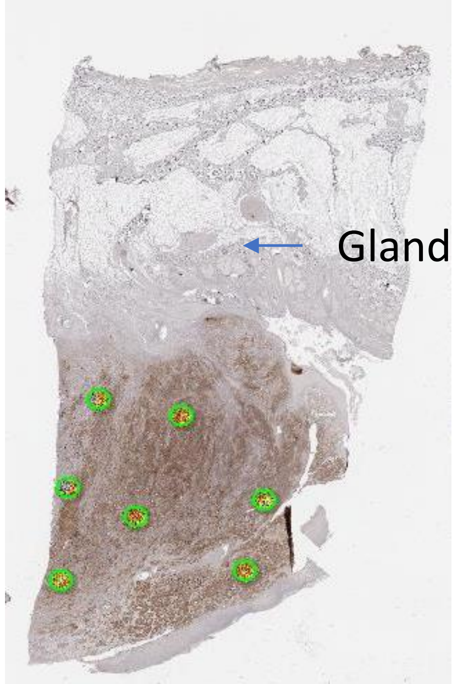

Glands

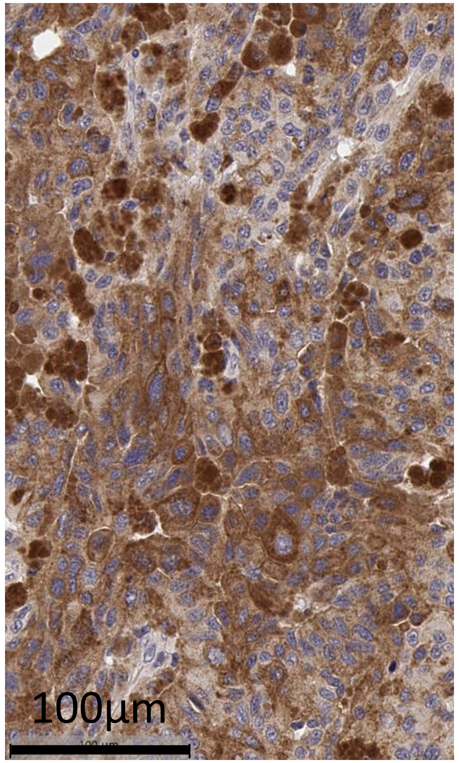

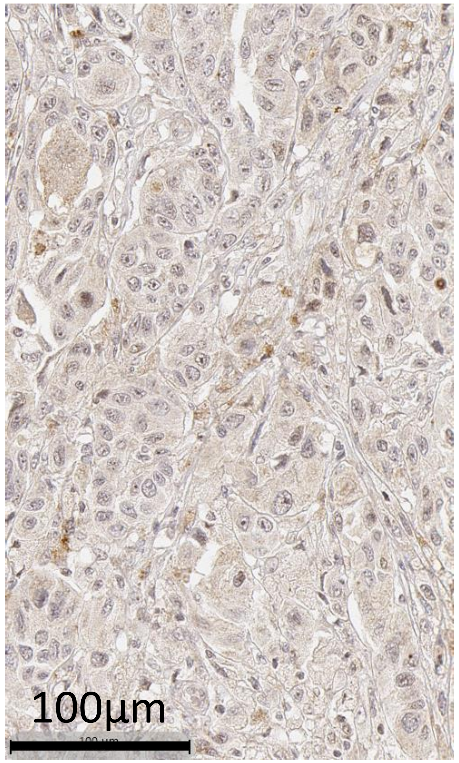

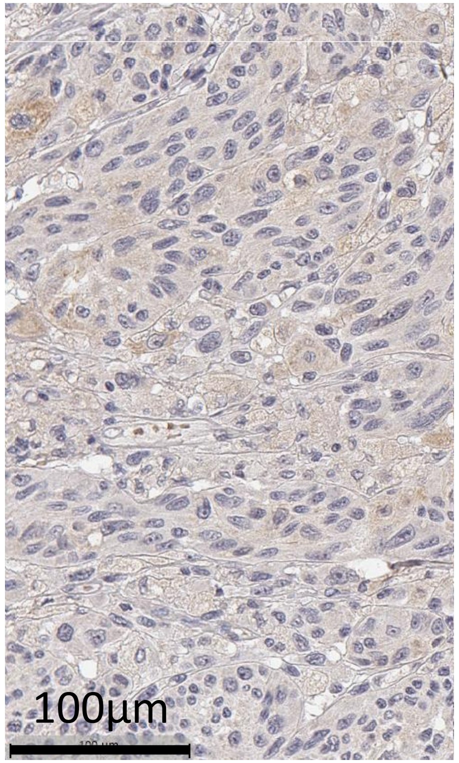

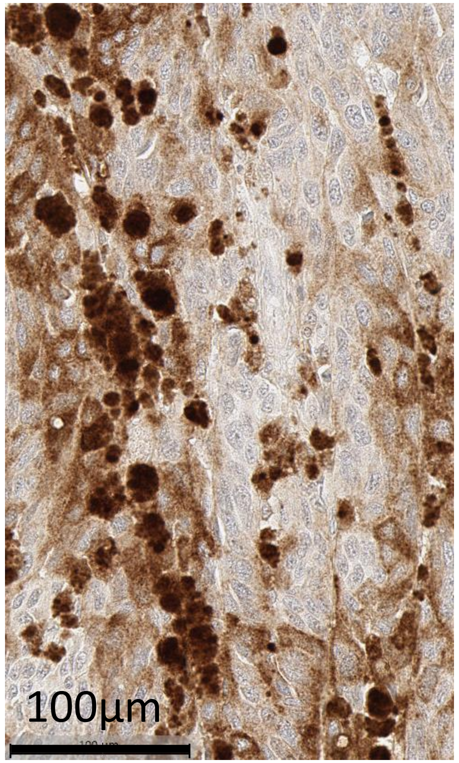

TAp53 cytoplasm high

Δ40p53 cytoplasm moderate

Infiltrating immune cells

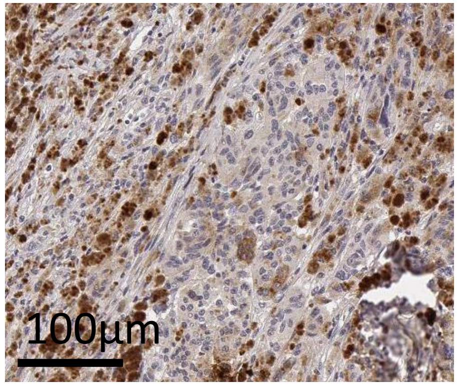

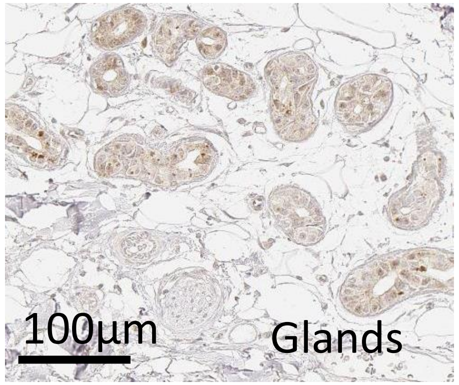

Q2020-064-S010
Primary melanoma
Slide: 126410, TAp53

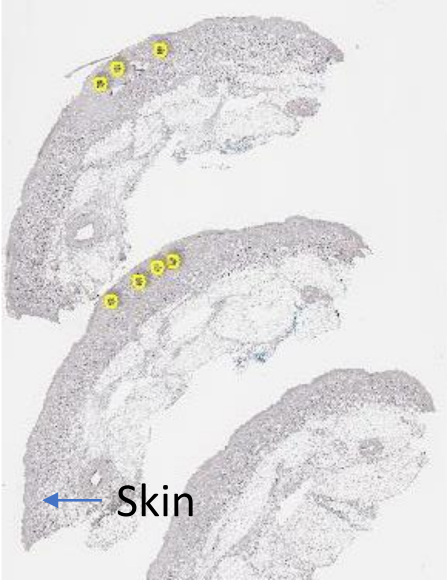

Δ40p53 nucleus moderate

TAp53 H-scores:
Cytoplasm: 11.42
Nucleus: 49.91

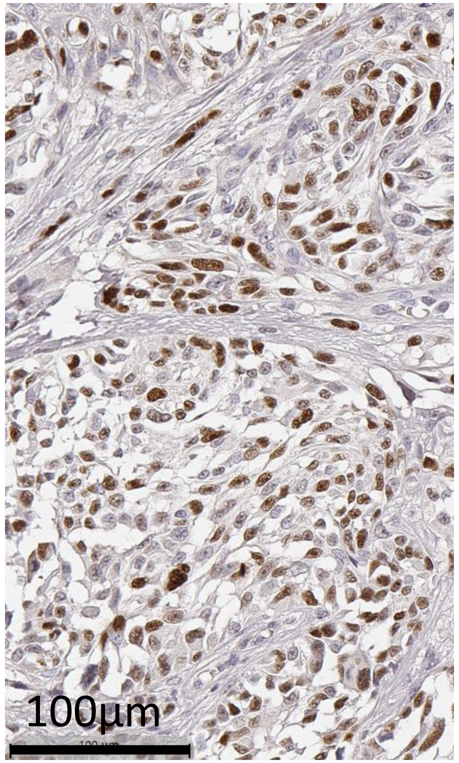

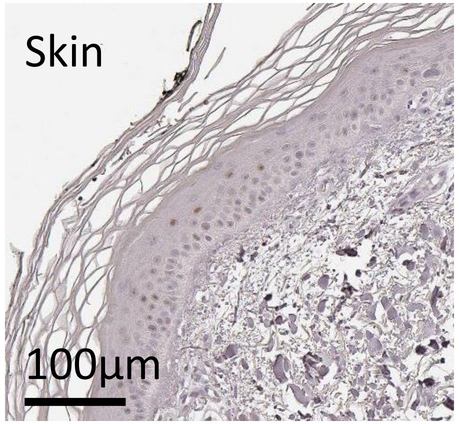

p53β H-scores:
Cytoplasm: 0.10
Nucleus: 55.99

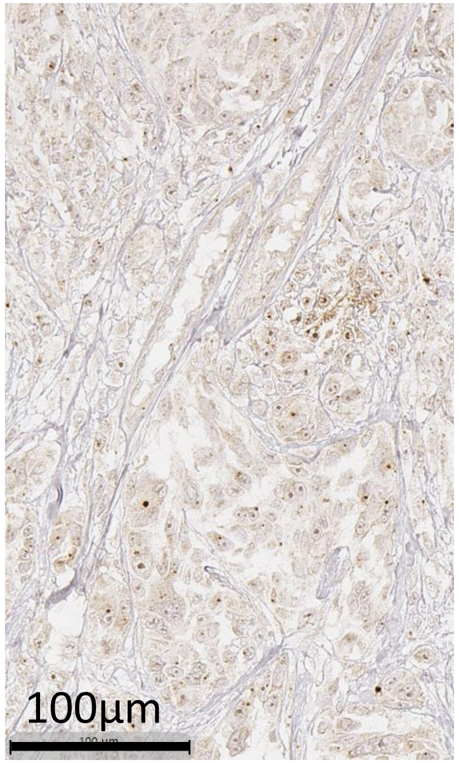

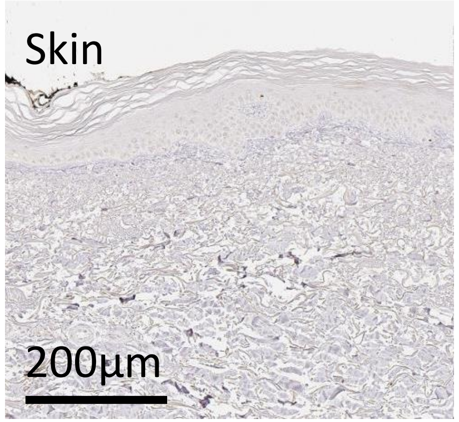

Δ133p53 H-scores:
Cytoplasm: 0.50
Nucleus: 7.13

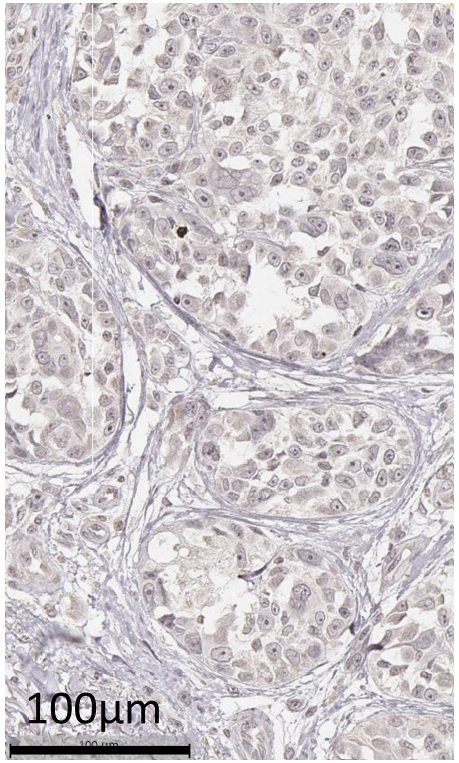

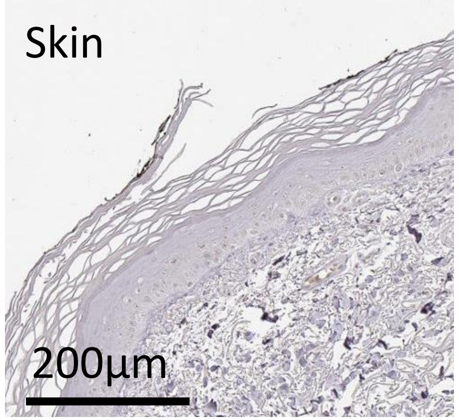

Δ40p53 H-scores:
Cytoplasm: 0.23
Nucleus: 59.82

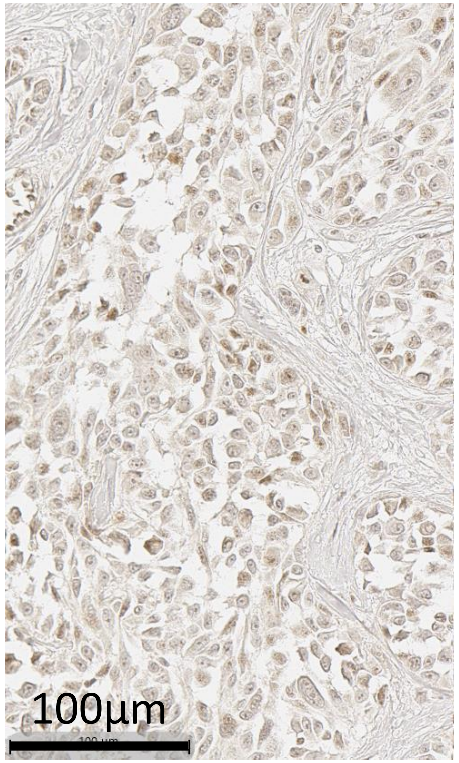

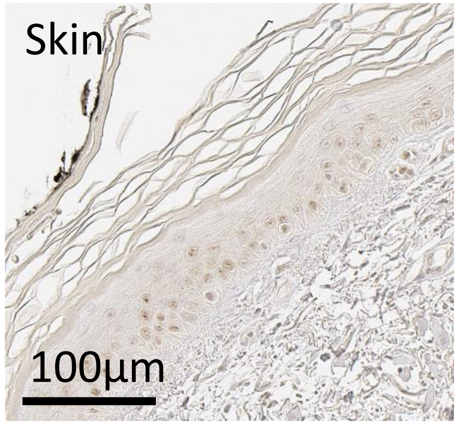

Q2020-064-S116
Metastatic melanoma
Lymph Node
Slide: 128246, TAp53

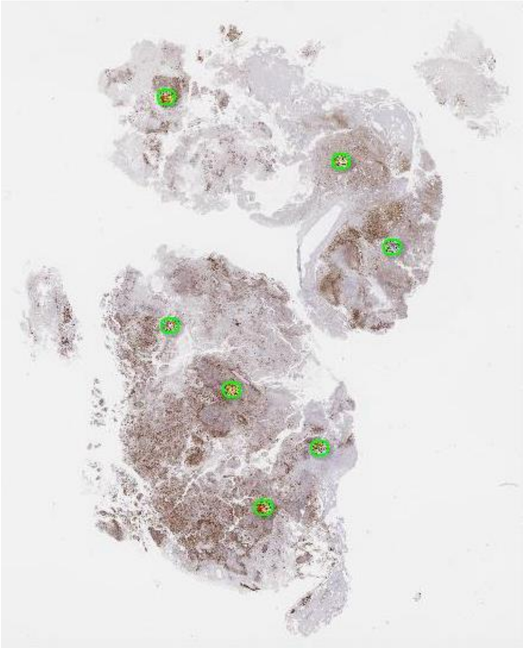

p53β cytoplasm high

Δ133p53 cytoplasm high

Δ40p53 cytoplasm high
Δ40p53 nucleus high

TAp53 H-scores:
Cytoplasm: 69.55
Nucleus: 115.30

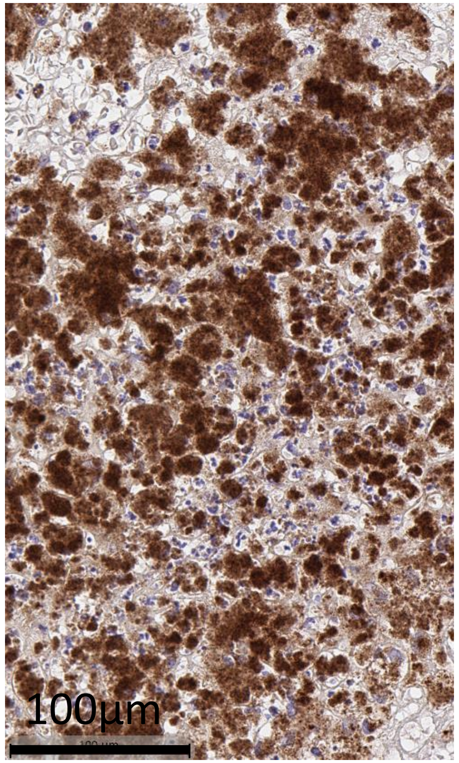

p53β H-scores:
Cytoplasm: 149.68
Nucleus: 210.65

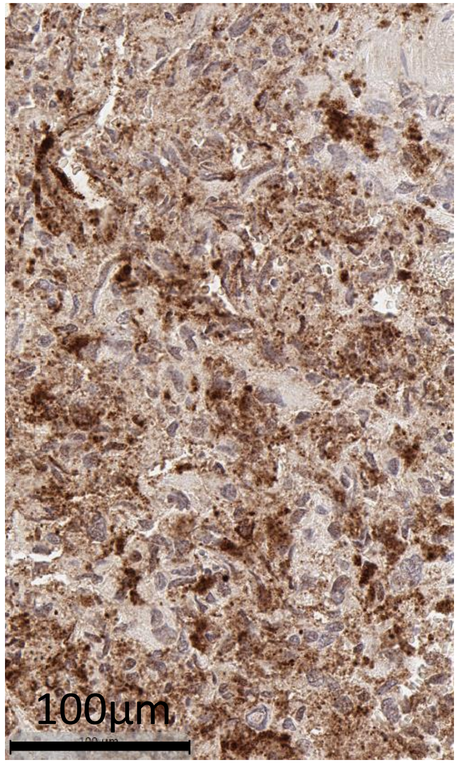

Δ133p53 H-scores:
Cytoplasm: 125.614
Nucleus: 209.44

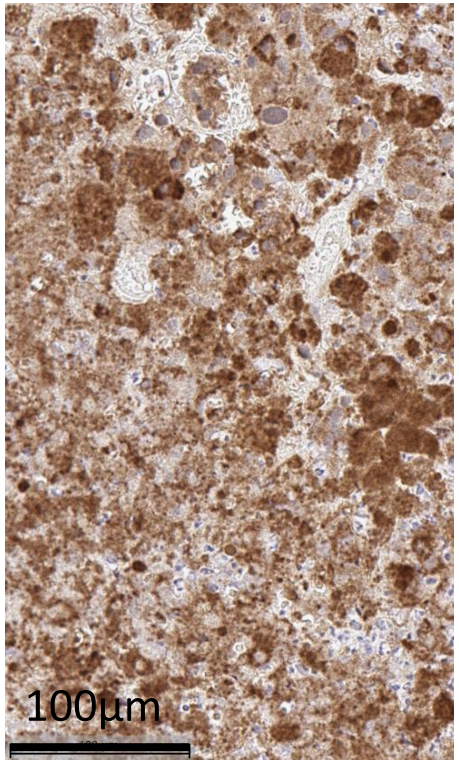

Δ40p53 H-scores:
Cytoplasm: 144.52
Nucleus: 232.81

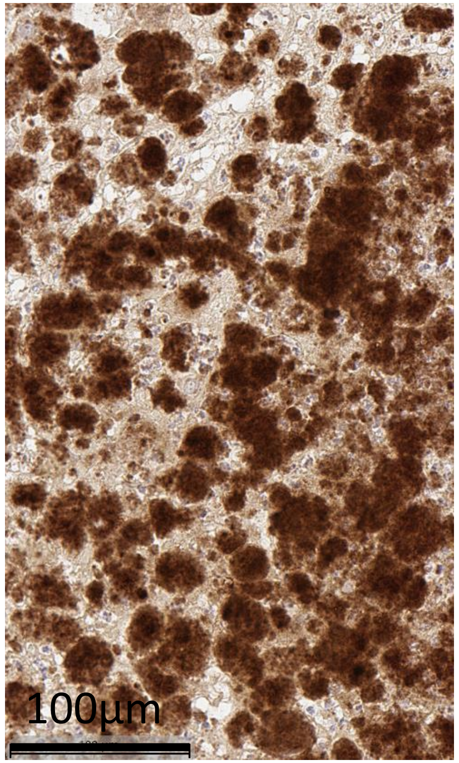

Q2020-064-S110
Metastatic melanoma
Brain
Slide: 126474, TAp53

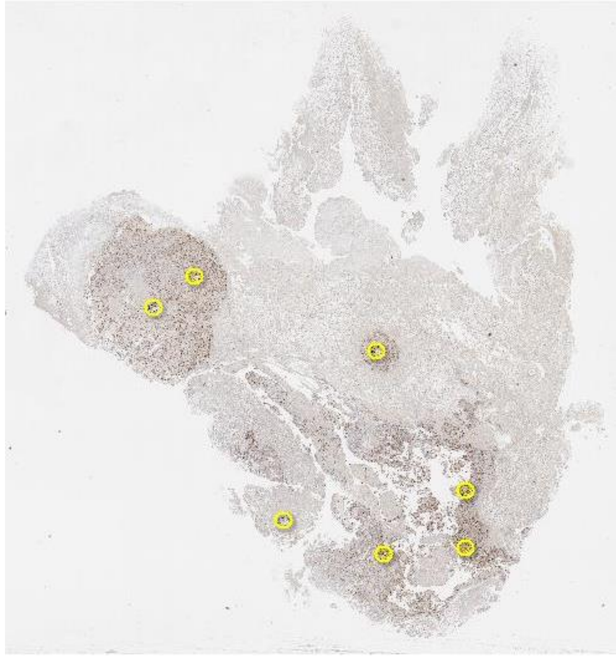

p53β nucleus high

Δ133p53 cytoplasm moderate

TAp53 H-scores:
Cytoplasm: 58.72
Nucleus: 139.38

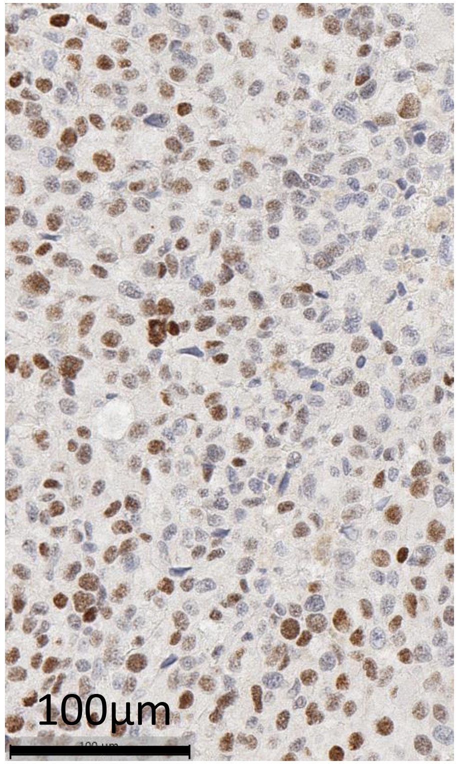

p53β H-scores:
Cytoplasm: 84.24
Nucleus: 151.04

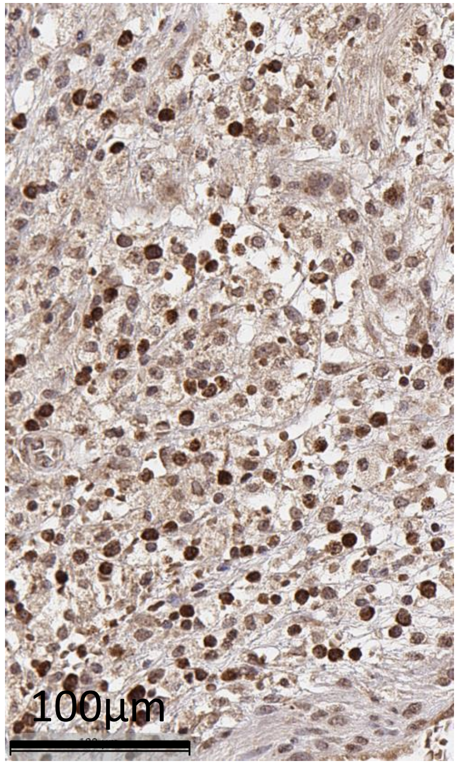

Δ133p53 H-scores:
Cytoplasm: 45.23
Nucleus: 80.87

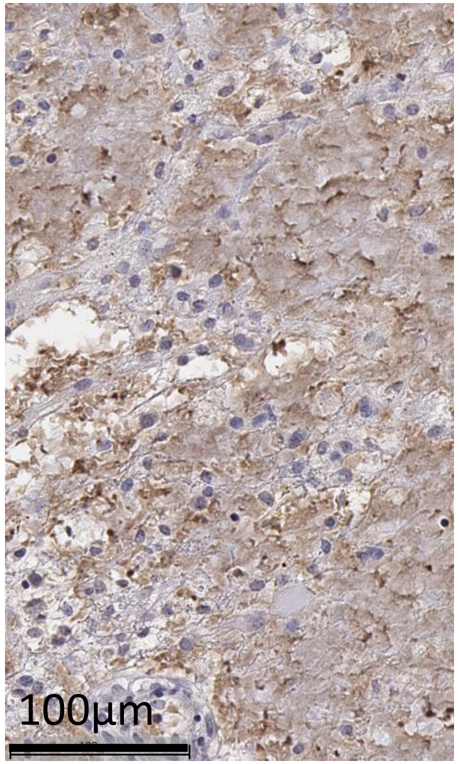

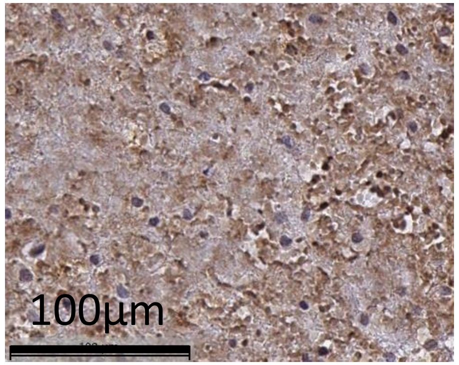

Δ40p53 H-scores:
Cytoplasm: 0.59
Nucleus: 27.42

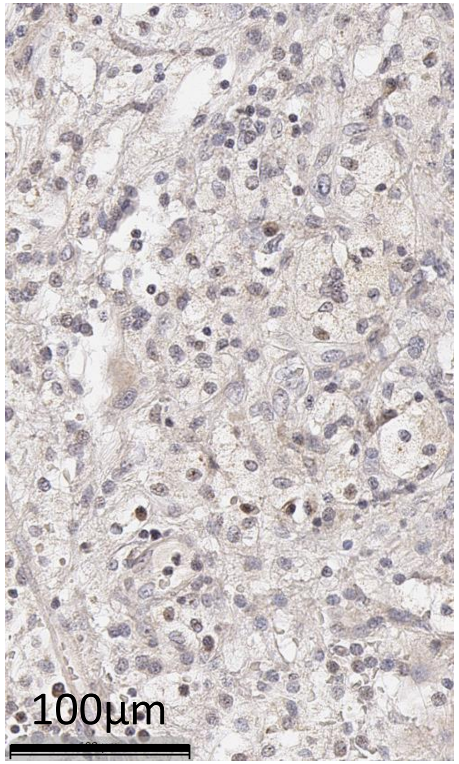

Q2020-064-S023
Primary melanoma
Slide: 126420, TAp53

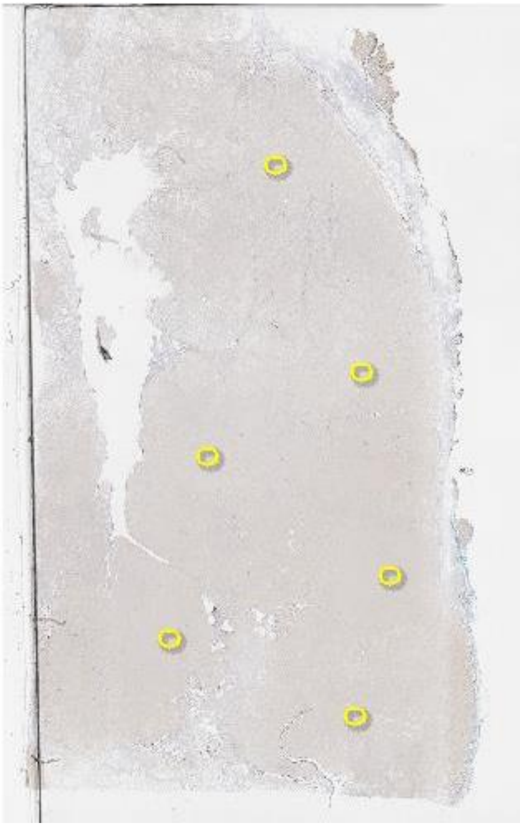

TAp53 H-scores:
Cytoplasm: 0.00
Nucleus: 10.74

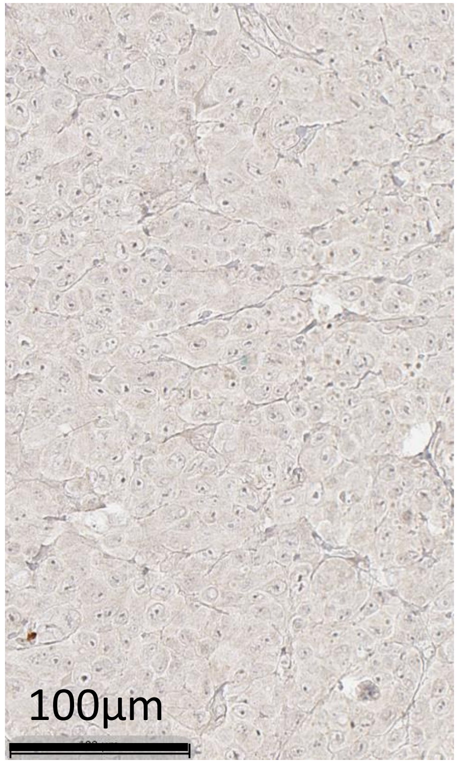

p53β H-scores:
Cytoplasm: 0.78
Nucleus: 37.45

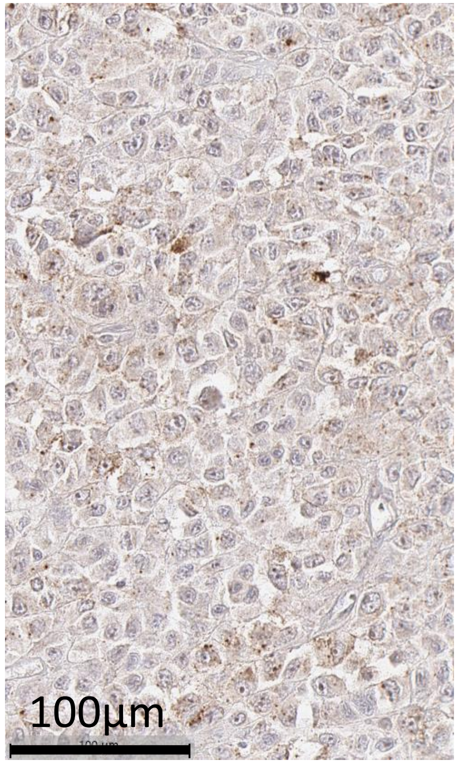

Δ133p53 H-scores:
Cytoplasm: 0.00
Nucleus: 0.36

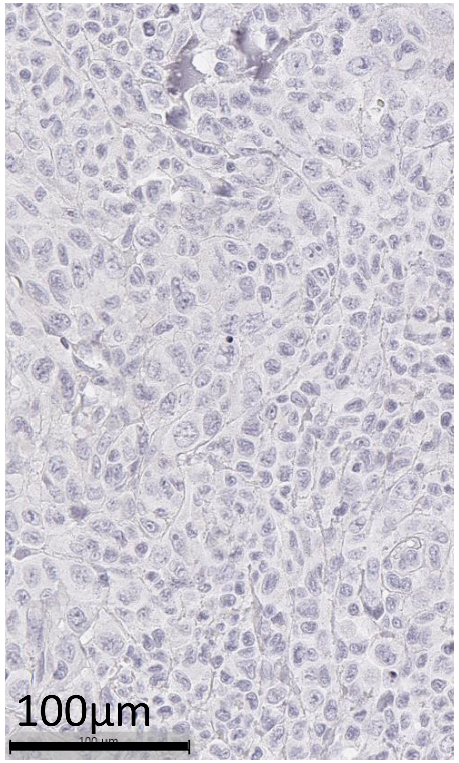

Δ40p53 H-scores:
Cytoplasm: 0.00
Nucleus: 0.53

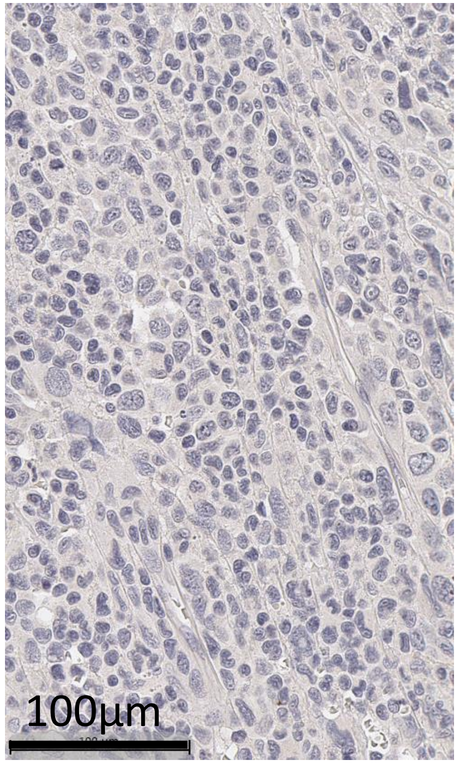

Δ133p53 cytoplasm low
Δ133p53 nucleus low

Q2020-064-S107
Primary melanoma
Slide: 126472, TAp53

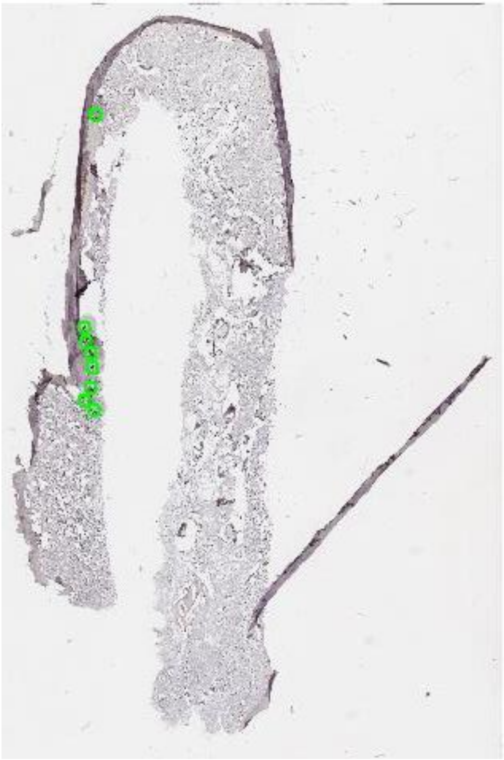

TAp53 H-scores:
Cytoplasm: 12.91
Nucleus: 43.15

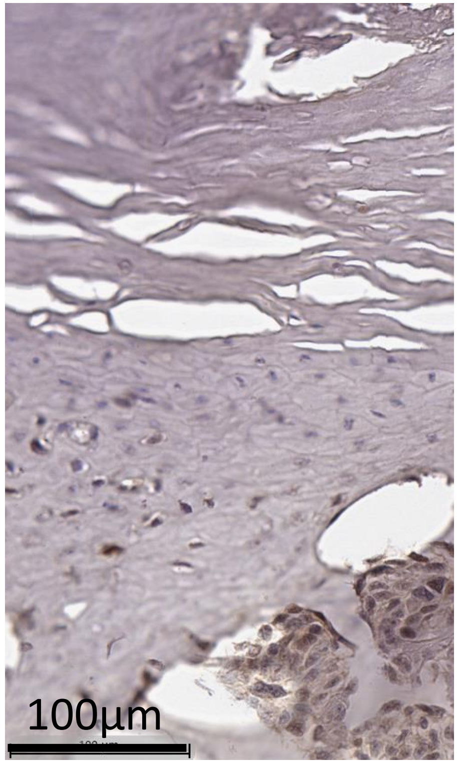

p53β H-scores:
Cytoplasm: 10.83
Nucleus: 58.22

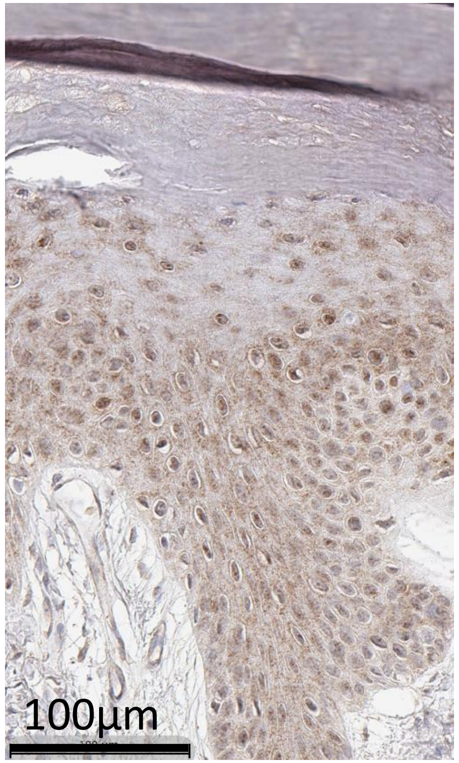

Δ133p53 H-scores:
Cytoplasm: 28.81
Nucleus: 55.58

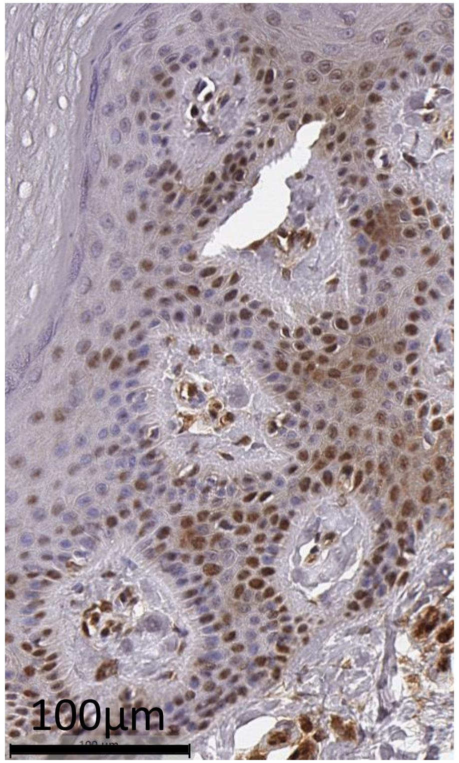

Δ40p53 H-scores:
Cytoplasm: 8.36
Nucleus: 32.04

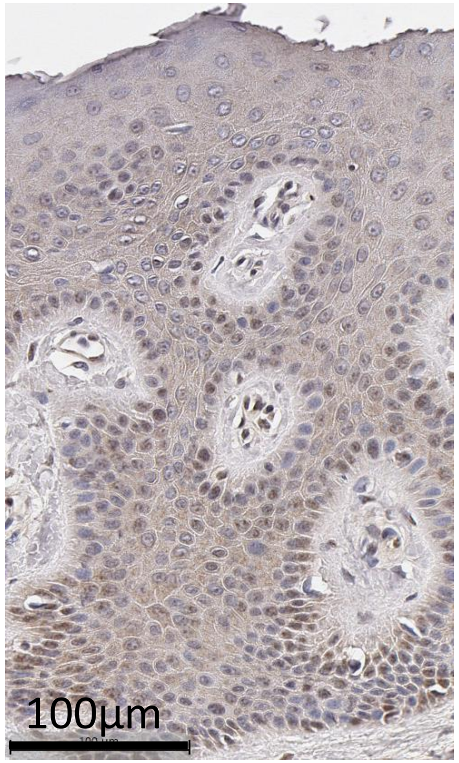

Δ133p53 nucleus moderate

Q2020-064-S119
Primary melanoma
Slide: 128243, TAp53

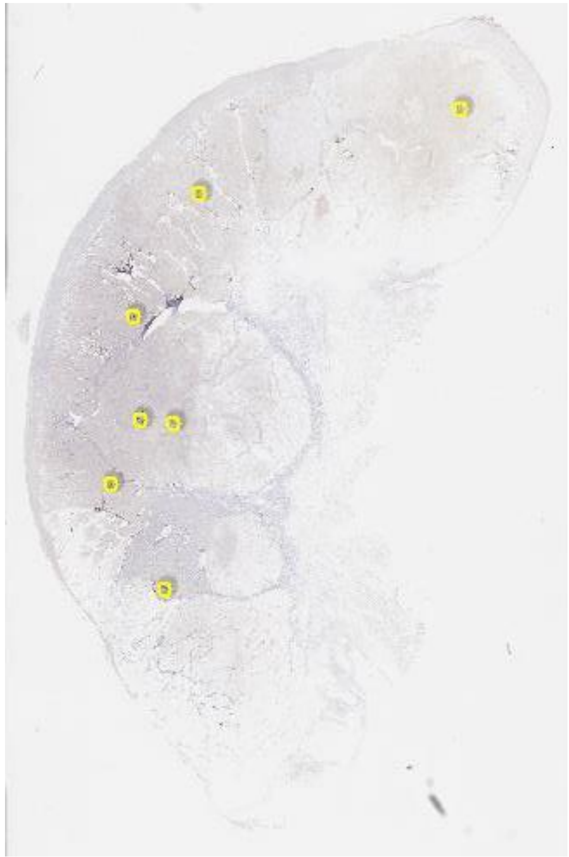

TAp53 H-scores:
Cytoplasm: 0.00
Nucleus: 7.15

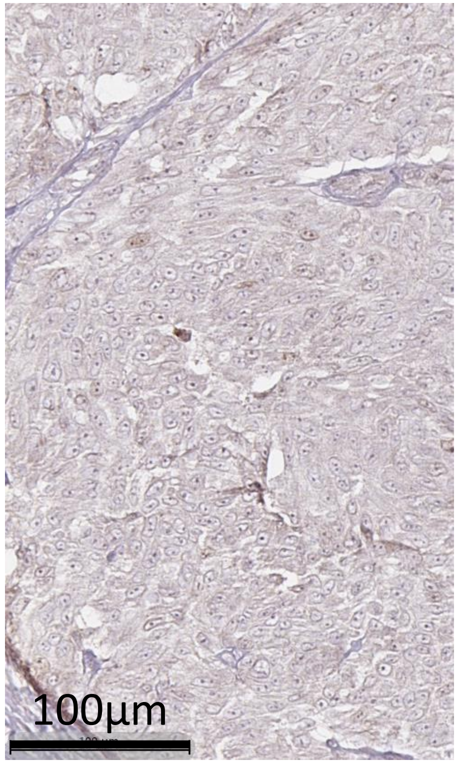

p53β H-scores:
Cytoplasm: 0.00
Nucleus: 3.13

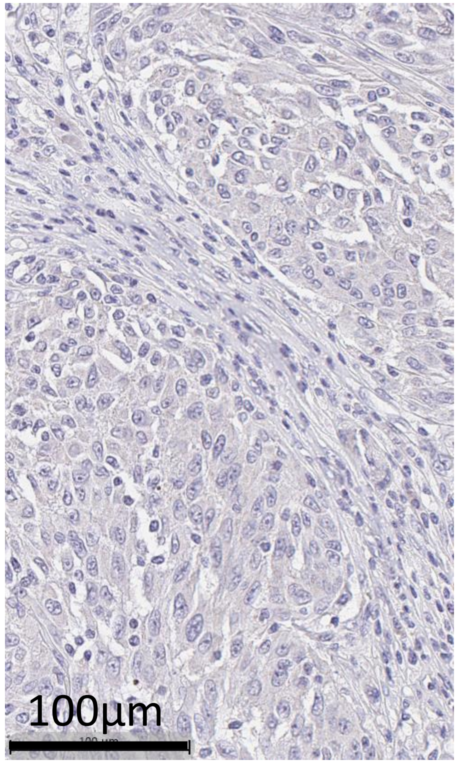

Δ133p53 H-scores:
Cytoplasm: 0.13
Nucleus: 3.86

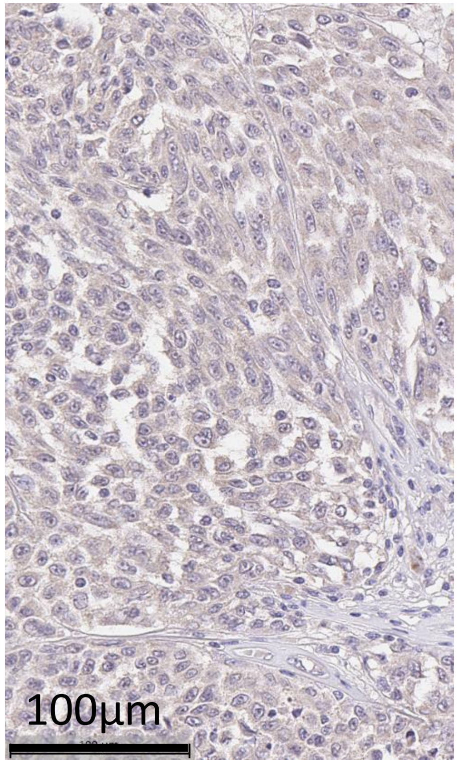

Δ40p53 H-scores:
Cytoplasm: 1.26
Nucleus: 6.07

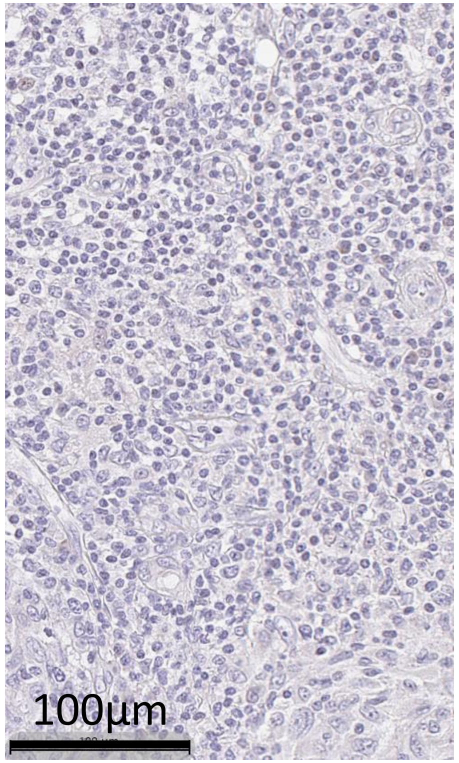

p53β cytoplasm low

Q2020-064-S090
Metastatic melanoma
Lymph Node
Slide: 126458, TAp53

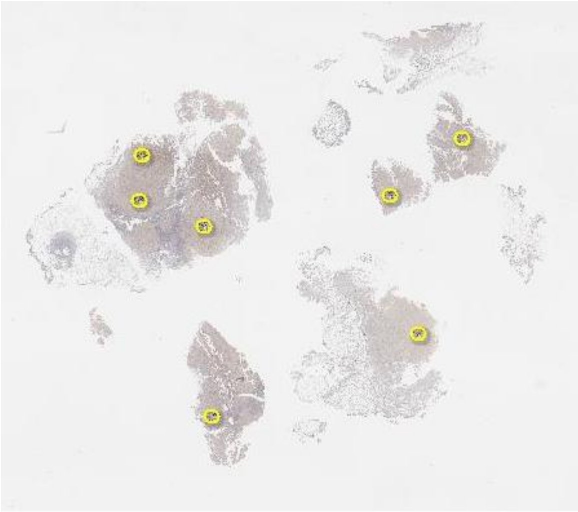

p53β cytoplasm
moderate

TAp53 H-scores:
Cytoplasm: 12.74
Nucleus: 65.34

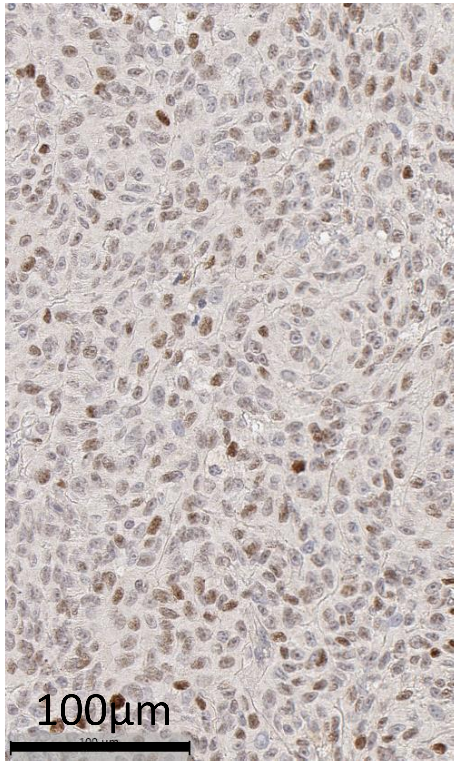

p53β H-scores:
Cytoplasm: 72.77
Nucleus: 94.67

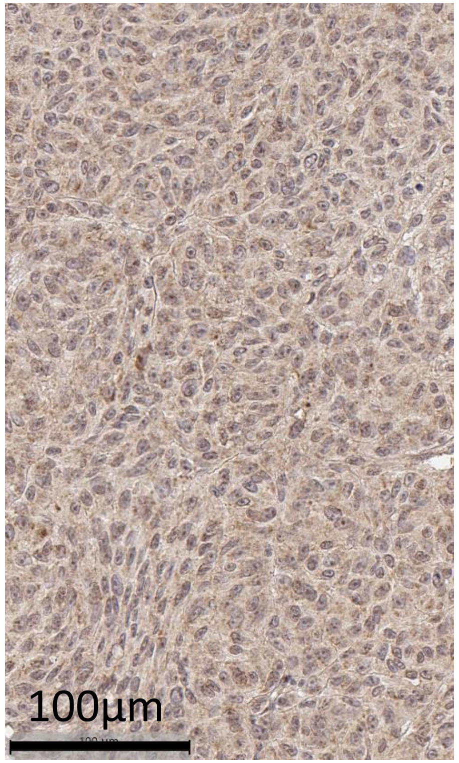

Δ133p53 H-scores:
Cytoplasm: 0.01
Nucleus: 3.42

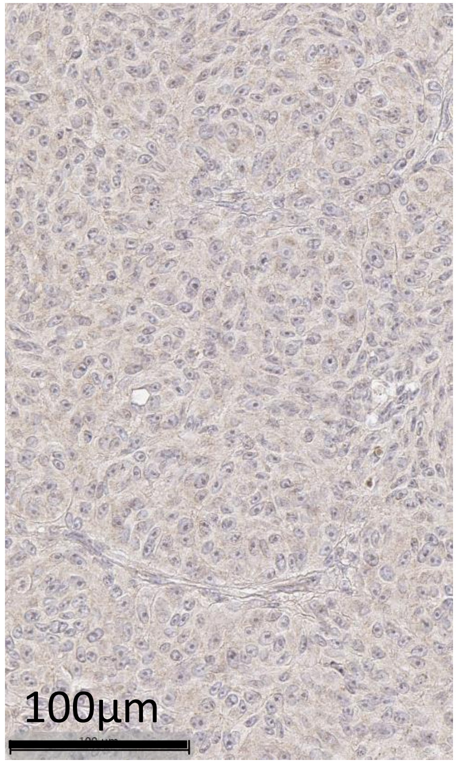

Δ40p53 H-scores:
Cytoplasm: 0.00
Nucleus: 0.46

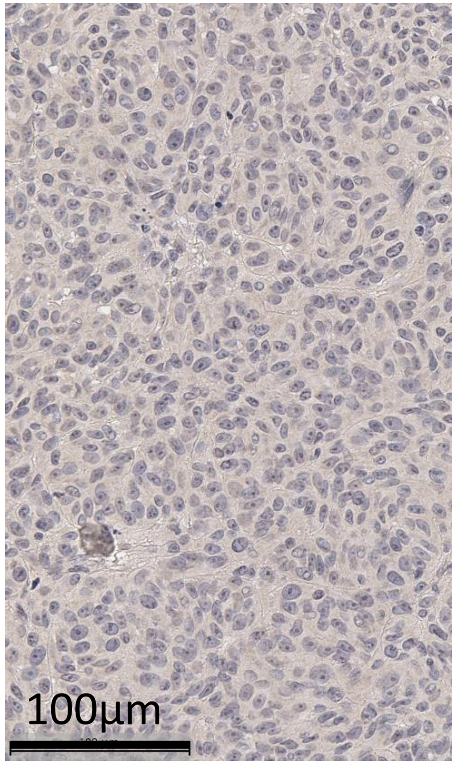

Q2020-064-S121
Primary melanoma
Slide: 128261, p53β

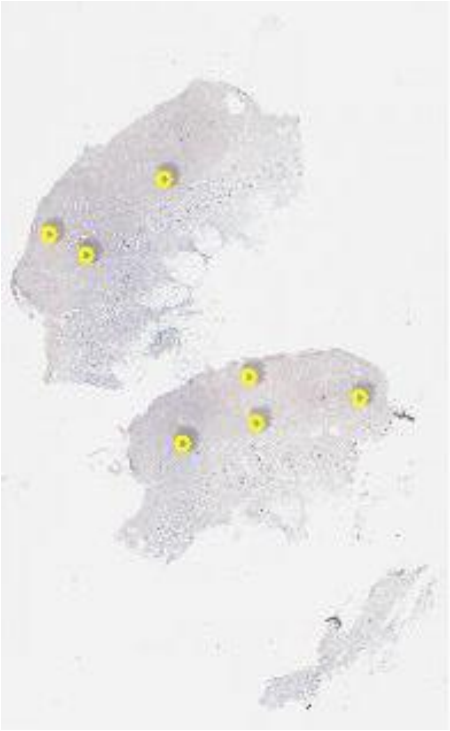

TAp53 H-scores:
Cytoplasm: N/A
Nucleus: N/A

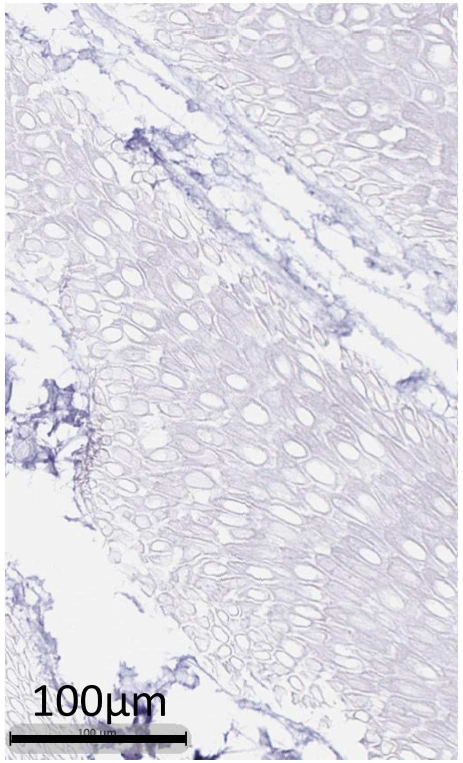

p53β H-scores:
Cytoplasm: 0.00
Nucleus: 2.15

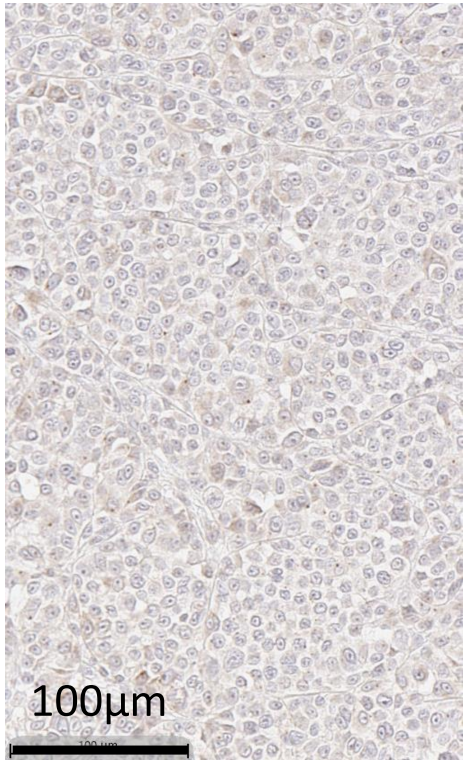

Δ133p53 H-scores:
Cytoplasm: 0.05
Nucleus: 0.64

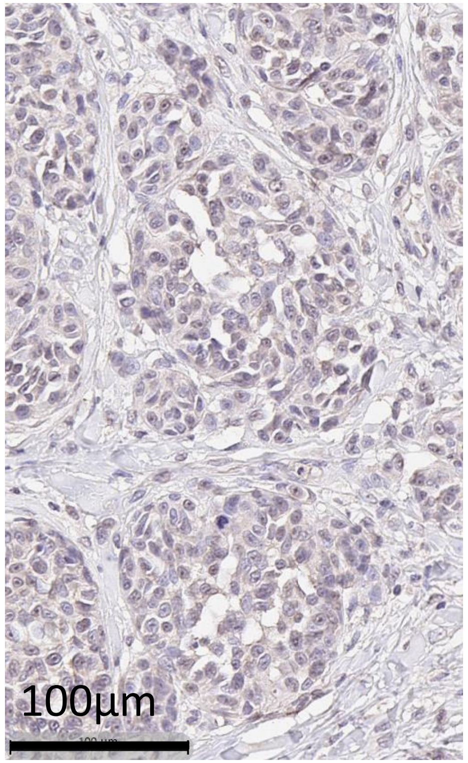

Δ40p53 H-scores:
Cytoplasm: 0.20
Nucleus: 1.64

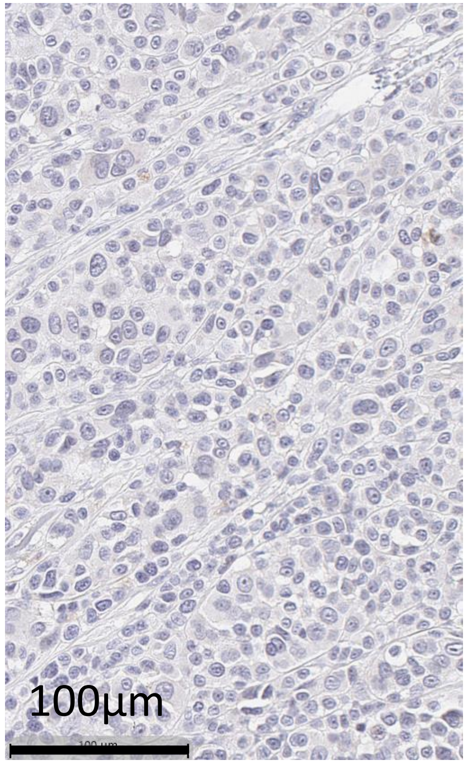

p53β nucleus low

Q2020-064-S046
Primary melanoma
Slide: 126431, TAp53

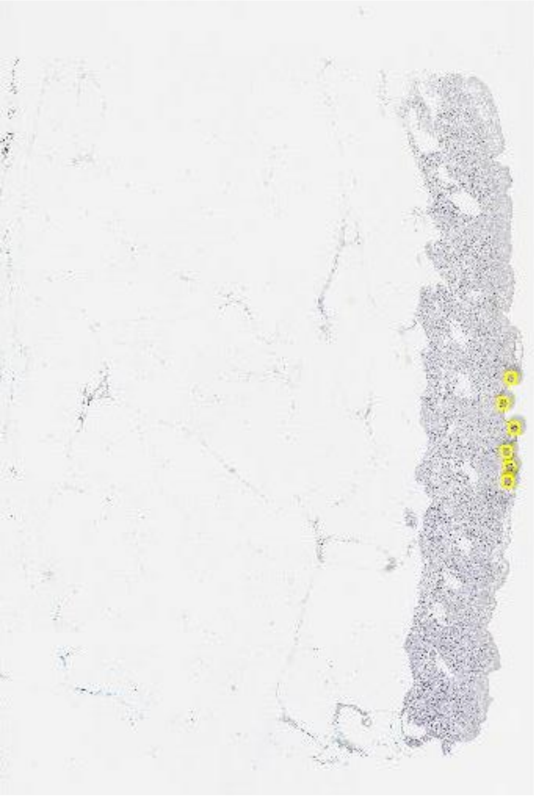

TAp53 H-scores:
Cytoplasm: 2.46
Nucleus: 22.16

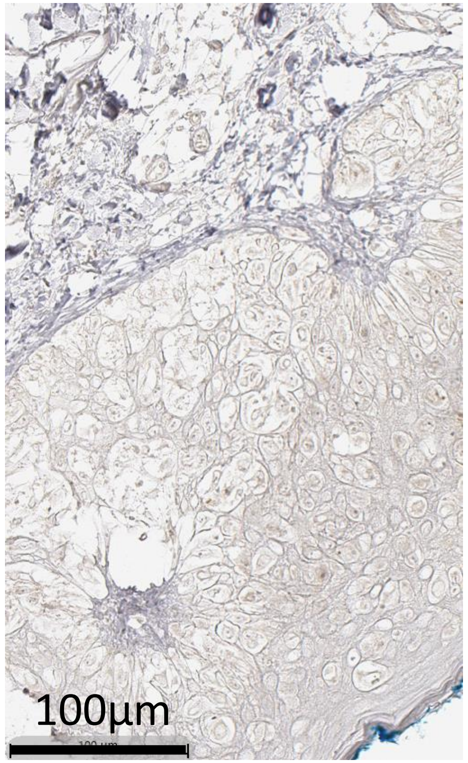

p53β H-scores:
Cytoplasm: 36.80
Nucleus: 96.27

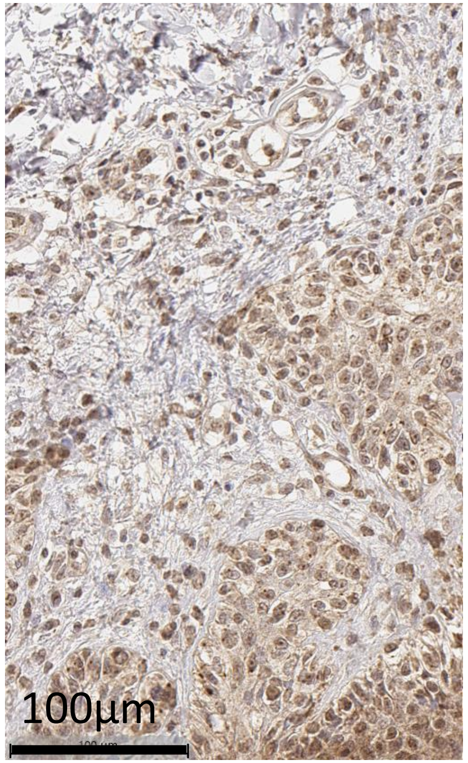

Δ133p53 H-scores:
Cytoplasm: 0.15
Nucleus: 0.97

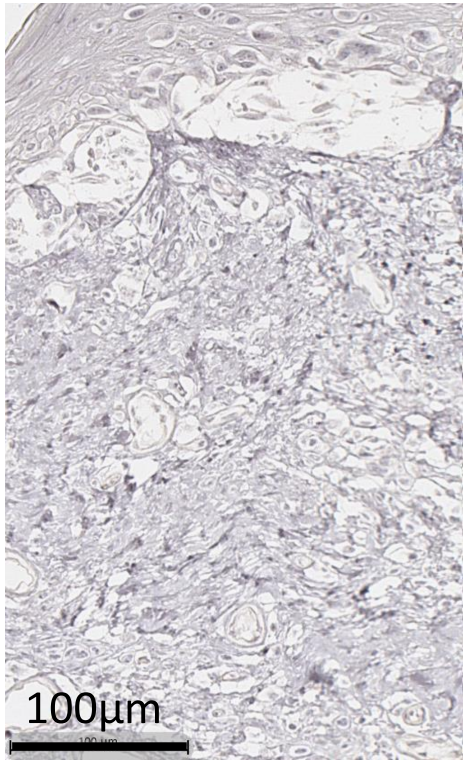

Δ40p53 H-scores:
Cytoplasm: 4.79
Nucleus: 69.07

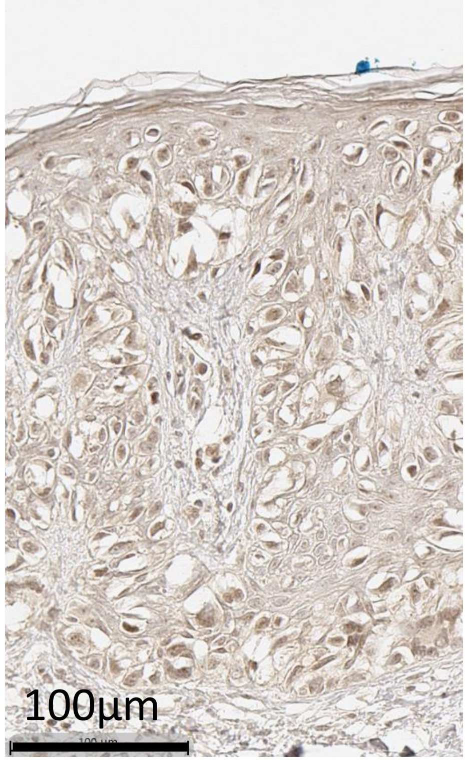

p53β nucleus moderate

Q2020-064-S102
Metastatic melanoma
Lymph node
Slide: 126468, TAp53

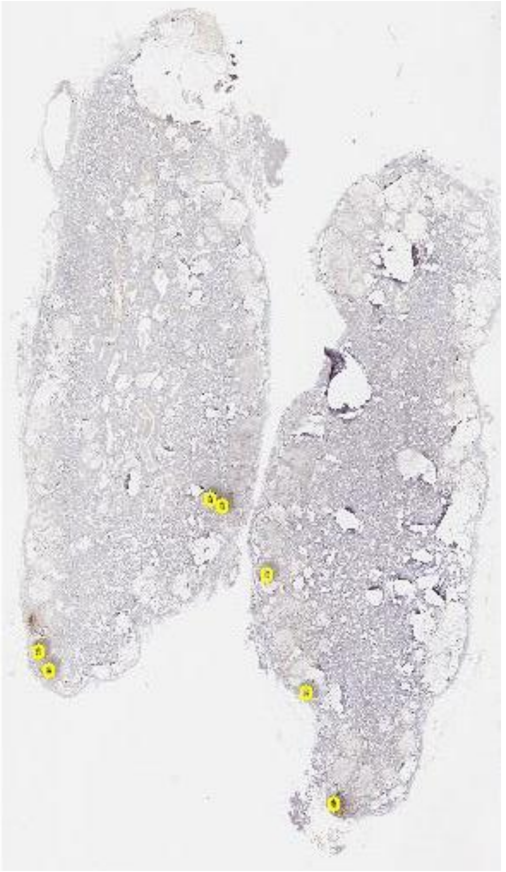

TAp53 H-scores:
Cytoplasm: 51.32
Nucleus: 107.13

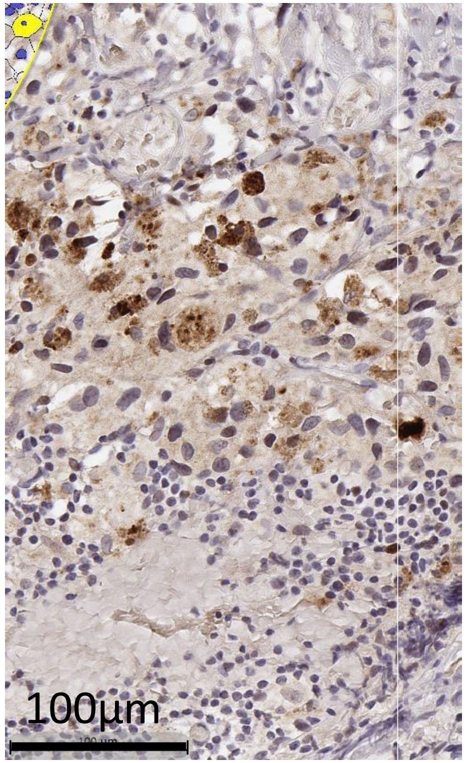

p53β H-scores:
Cytoplasm: 4.96
Nucleus: 71.73

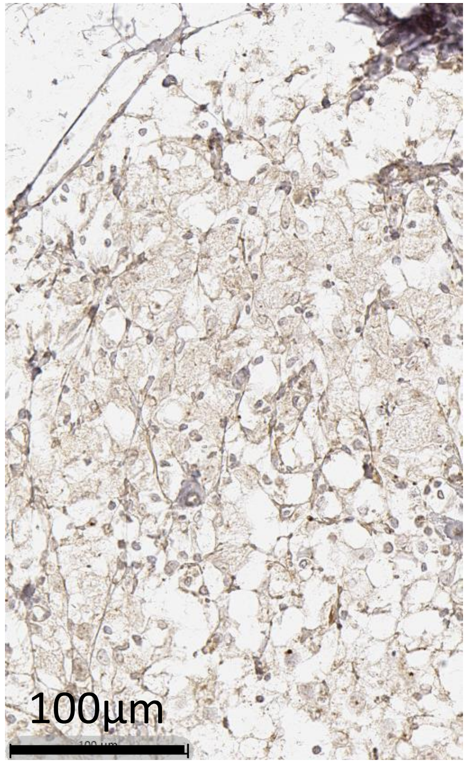

Δ133p53 H-scores:
Cytoplasm: 29.99
Nucleus: 54.22

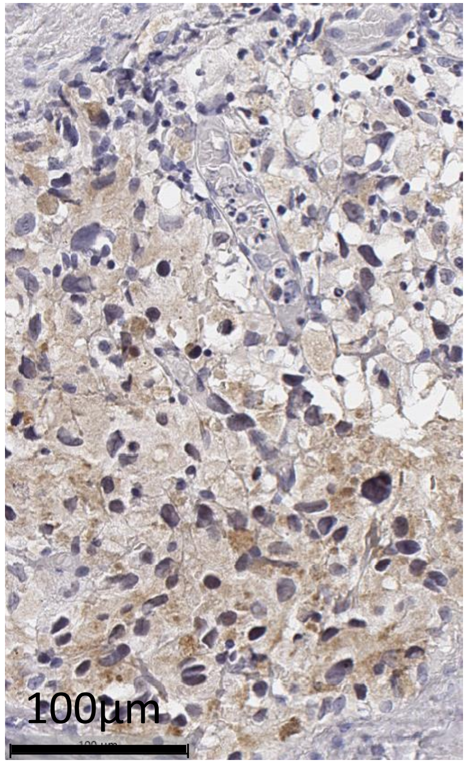

Δ40p53 H-scores:
Cytoplasm: 4.47
Nucleus: 54.32

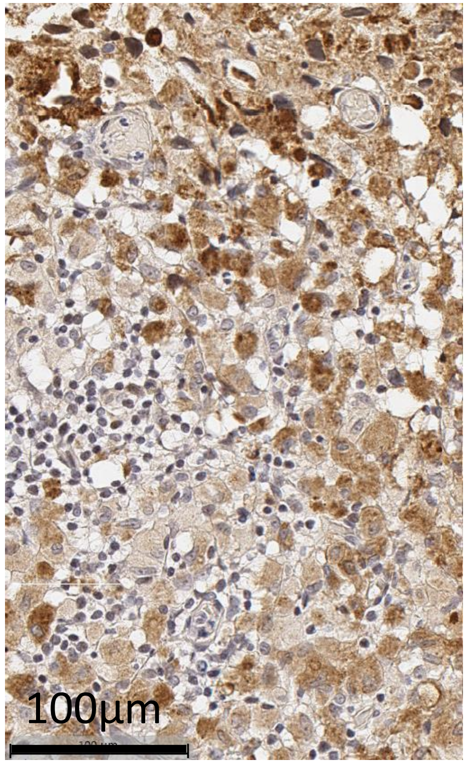

TAp53 cytoplasm
moderate

Q2020-064-S031
Primary melanoma
Slide: 126468, TAp53

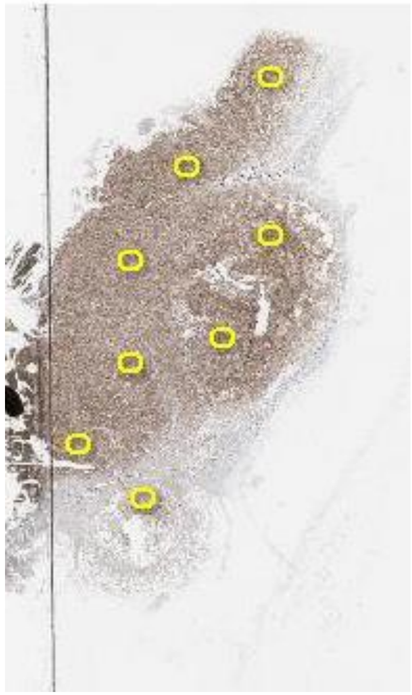

TAp53 H-scores:
Cytoplasm: 129.29
Nucleus: 223.78

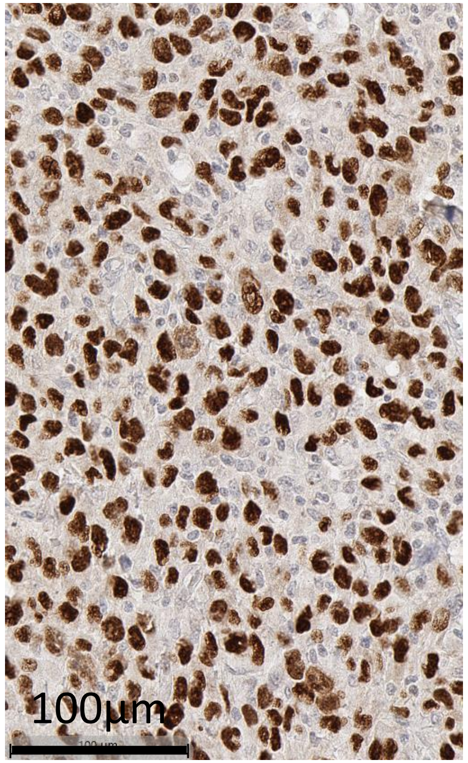

p53β H-scores:
Cytoplasm: 2.35
Nucleus: 95.96

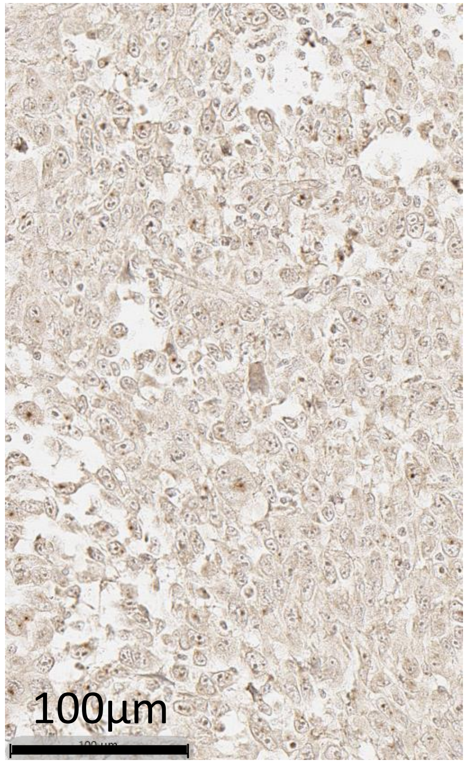

Δ133p53 H-scores:
Cytoplasm: 0.09
Nucleus: 3.37

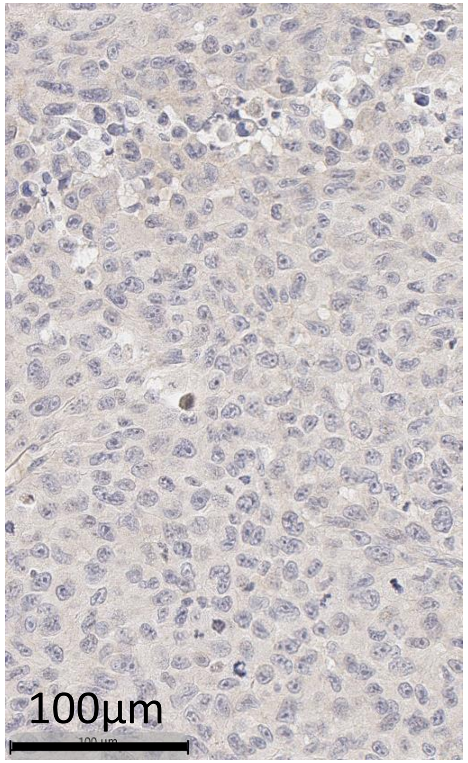

Δ40p53 H-scores:
Cytoplasm: 0.12
Nucleus: 6.65

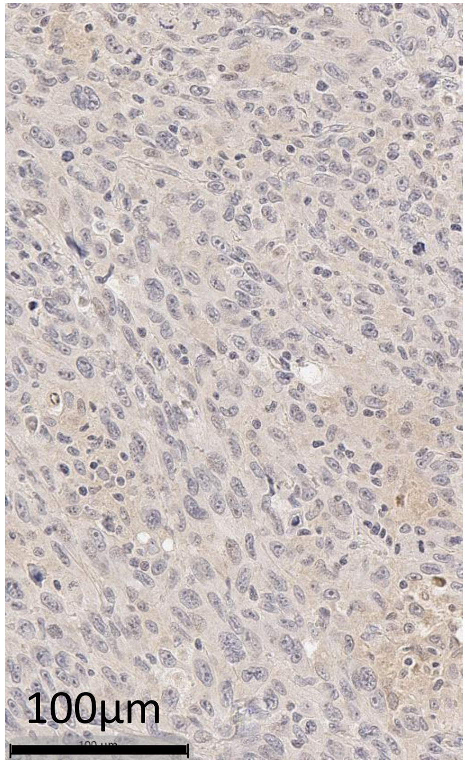

TAp53 nucleus high

Q2020-064-S083
Metastatic melanoma
Skin
Slide: 126454, TAp53

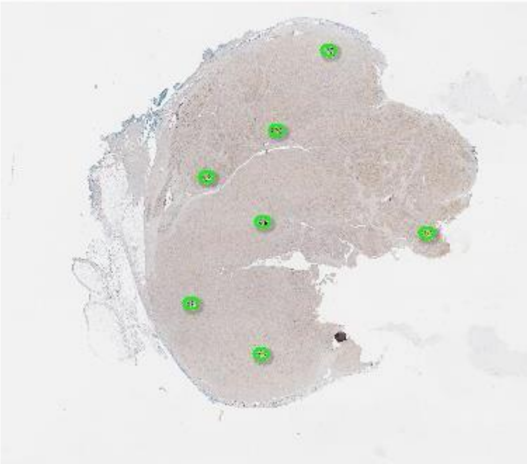

TAp53 nucleus
moderate

TAp53 H-scores:
Cytoplasm: 17.84
Nucleus: 89.14

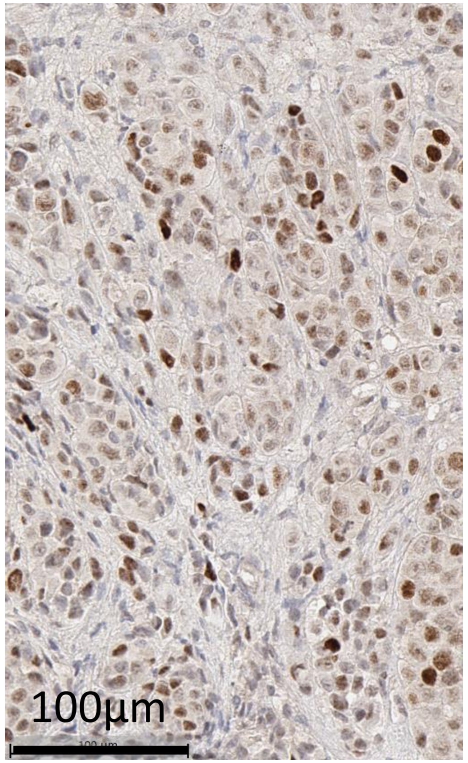

p53β H-scores:
Cytoplasm: 0.48
Nucleus: 41.61

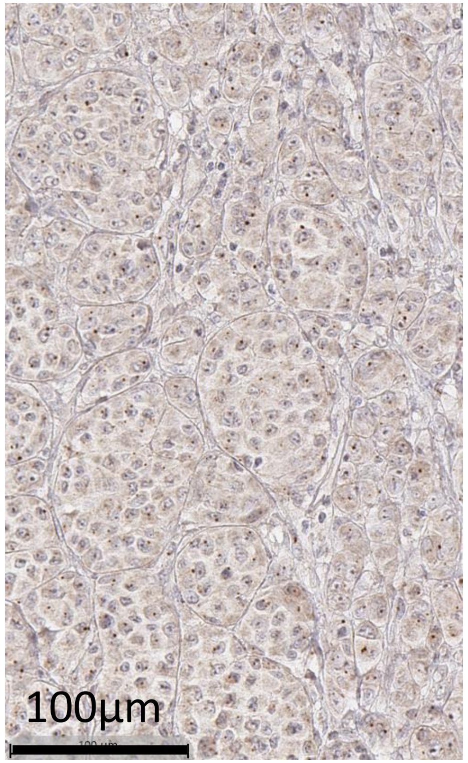

Δ133p53 H-scores:
Cytoplasm: 0.00
Nucleus: 3.57

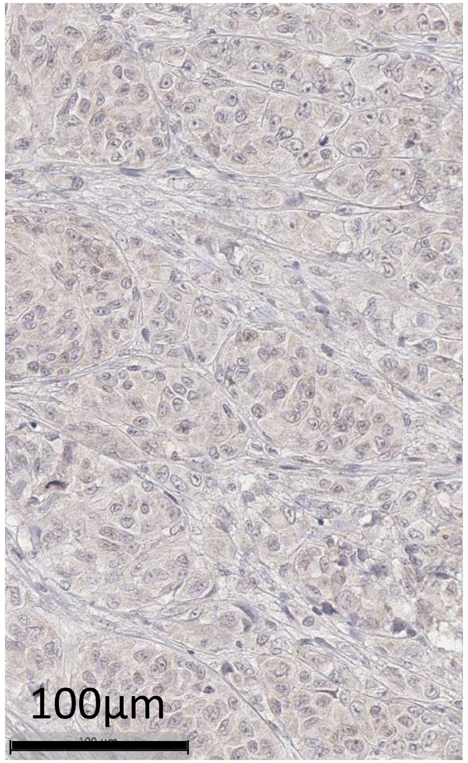

Δ40p53 H-scores:
Cytoplasm: 0.00
Nucleus: 0.84

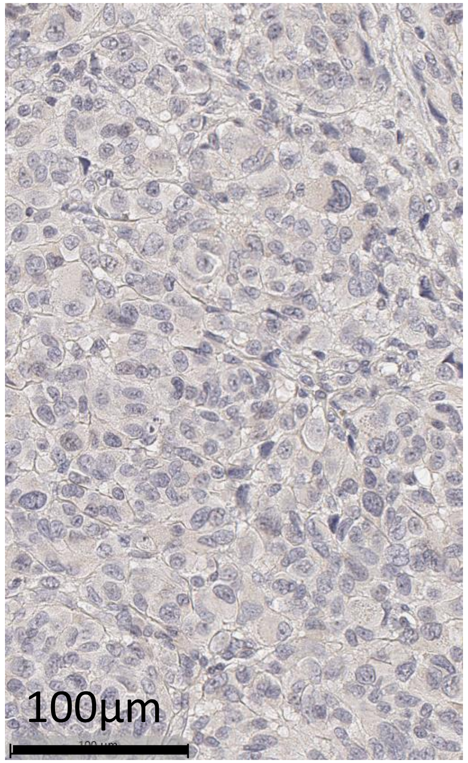

Supplement: Supplementary file 5 — Additional file 5: Representative annotations of immunostained slides of melanoma specimens. [file 12935_2023_3083_MOESM5_ESM.pdf]
